# Biocontrol of *Fusarium* head blight in rice using *Bacillus velezensis* JCK-7158

**DOI:** 10.3389/fmicb.2024.1358689

**Published:** 2024-06-10

**Authors:** Yu Jeong Yeo, Ae Ran Park, Bien Sy Vuong, Jin-Cheol Kim

**Affiliations:** ^1^Department of Agricultural Chemistry, Institute of Environmentally Friendly Agriculture, College of Agriculture and Life Science, Chonnam National University, Gwangju, Republic of Korea; ^2^Plant Healthcare Research Institute, JAN153 Biotech Incorporated, Gwangju, Republic of Korea

**Keywords:** *Fusarium* head blight, *Bacillus velezensis*, induced resistance, antifungal activity, biocontrol agent

## Abstract

*Fusarium* head blight (FHB) is a destructive disease caused by several species of *Fusarium*, such as *Fusarium graminearum* and *F. asiaticum*. FHB affects cereal crops, including wheat, barley, and rice, worldwide. *Fusarium*-infected kernels not only cause reduced yields but also cause quality loss by producing mycotoxins, such as trichothecenes and zearalenone, which are toxic to animals and humans. For decades, chemical fungicides have been used to control FHB because of their convenience and high control efficacy. However, the prolonged use of chemical fungicides has caused adverse effects, including the emergence of drug resistance to pathogens and environmental pollution. Biological control is considered one of the most promising alternatives to chemicals and can be used for integrated management of FHB due to the rare possibility of environment pollution and reduced health risks. In this study, *Bacillus velezensis* JCK-7158 isolated from rice was selected as an ecofriendly alternative to chemical fungicides for the management of FHB. JCK-7158 produced the extracellular enzymes protease, chitinase, gelatinase, and cellulase; the plant growth hormone indole-3-acetic acid; and the 2,3-butanediol precursor acetoin. Moreover, JCK-7158 exhibited broad antagonistic activity against various phytopathogenic fungi and produced iturin A, surfactin, and volatile substances as active antifungal compounds. It also enhanced the expression of *PR1*, a known induced resistance marker gene, in transgenic *Arabidopsis* plants expressing β-glucuronidase (GUS) fused with the *PR1* promoter. Under greenhouse conditions, treatments with the culture broth and suspension concentrate formulation of JCK-7158 at a 1,000-fold dilution inhibited the development of FHB by 50 and 66%, respectively. In a field experiment, treatment with the suspension concentrate formulation of JCK*-*7158 at a 1,000-fold dilution effectively controlled the development of FHB with a control value of 55% and reduced the production of the mycotoxin nivalenol by 40%. Interestingly, treatment with JCK-7158 enhanced the expression of plant defense-related genes in salicylic acid, jasmonic acid, ethylene, and reactive oxygen species (ROS) signaling pathways before and after FHB pathogen inoculation. Taken together, our findings support that JCK-7158 has the potential to serve as a new biocontrol agent for the management of FHB.

## 1 Introduction

*Fusarium* head blight (FHB) is a significant disease affecting wheat, barley, rice, and other grain crops worldwide (Goswami and Kistler, 2004; McMullen et al., 2012). The primary causative pathogens of FHB belong to the *Fusarium graminearum* species complex (FGSC), which includes *F. asiaticum* and *F. graminearum* (Goswami and Kistler, 2005; Starkey et al., 2007). When several *Fusarium* species infest the grain, fungal colonies develop within the grain and disrupt nutrient supply to the top. This has a detrimental impact on grain size, weight, and germination rate, ultimately reducing grain yield and quality (Parry et al., 1995; Champeil et al., 2004). Furthermore, FHB triggers the production of *Fusarium* mycotoxins, such as deoxynivalenol (DON), nivalenol (NIV), and zearalenone (ZEA), which pose serious health risks to humans and livestock (Peraica et al., 1999; Pieters et al., 2002). *Fusarium* mycotoxins can cause dermal toxicity, alimentary toxic aleukia, food refusal, vomiting, and depressed immune functions (Snijders and Perkowski, 1990; Palazzini et al., 2007).

Various methods have been used to control FHB; these include the use of healthy seeds, cultivation methods, rotation, application of fungicides, and breeding of resistant varieties. However, none of these strategies have proven effective in completely eradicating the disease because FHB lacks genetic resistance, the inoculum is distributed over long distances and is ubiquitous, and the range of pathogen hosts is wide (Snyder and Nash, 1968; Milus and Parsons, 1994; Dill-Macky and Jones, 2000; Matarese et al., 2012; Reis and Carmona, 2013). Moreover, long-term chemical use has led to numerous problems, such as increased pathogen resistance, chemical residues, environmental pollution, and health risks. At present, given its rare contamination potential and reduced health risks, biocontrol is regarded as one of the most promising alternatives to chemicals and can be used for the integrated management of FHB.

Various antagonistic bacteria have been considered as potential biocontrol agents. These bacteria inhibit diseases through various mechanisms, including competition, the production of hydrolases, and the production of antimicrobials and volatile organic compounds (VOCs) (Elad and Baker, 1985; Senthilkumar et al., 2009). Moreover, they enhance plant disease resistance by synthesizing various hormones, such as indole-3-acetic acid (IAA) and gibberellin, or by decomposing various organic substances, such as chitin and cellulose, which are components of fungal cell walls (Idris et al., 2007). Among the various antagonistic bacteria, several species belonging to the genus *Bacillus* have distinct advantages over those belonging to other genera of antagonistic bacteria.

*Bacillus* is a gram-positive bacterium belonging to the phylum Firmicutes. It forms rod-shaped endospores; therefore, it is resistant to heat, ultraviolet (UV) light, and drying (Schallmey et al., 2004; Logan et al., 2009). It has excellent advantages over other genera such as *Trichoderma* and *Pseudomonas* for manufacturing formulations and facilitating production and storage for longer periods by producing endospores (Santoyo et al., 2012).

*Bacillus velezensis* is a species that has 99% similarity in 16 s rRNA gene sequence to *Bacillus amyloliquefaciens* and is found in several environments, some of which live as plant endophytes (Ye et al., 2018; Liang et al., 2023). *Bacillus velezensis* produces various secondary metabolites like cyclic lipopeptides (Ongena and Jacques, 2008). *B. velezensis* and its molecules have inhibited the growth of *F. graminearum in vitro* and controlled FHB in wheat (Palazzini et al., 2016; Chen et al., 2018; Palazzini et al., 2022). To our best knowledgement, all of the papers described that *B. velezensis* suppressed the development of FHB by action mechanism of antifungal activity, but not by action mechanism of induced resistance. However, it has been reported to exhibit induced resistance by activating the immune function of the plant against fungal and viral diseases (Jiang et al., 2019; Abdelkhalek et al., 2020).

Induced resistance is classified into systemic acquired resistance (SAR), which is induced by signal transmitters of salicylic acid (SA), nonpathogens, pathogens, or specific substances present in nature, and induced systemic resistance (ISR), which is induced by signal transmitters of jasmonic acid (JA) and ethylene (ET) (Yan et al., 2002). Induced resistance is a state in which disease resistance is physiologically increased in a plant by biotic or abiotic factors. The plant then activates its defense mechanism to resist disease. Such induced resistance is effective against diseases caused by a wide range of fungi, bacteria, and viruses. There is no induction of chemical resistance against plant diseases and pests, and substances produced by plants expressing induced resistance do not harm human health (Oostendorp et al., 2001; Choudhary et al., 2016).

To develop a biocontrol agent that can effectively control FHB through direct as well as indirect antagonism, JCK-7158, which exhibits antifungal activity against various phytopathogenic fungi and induces resistance, was selected from 606 strains isolated from rice in this study. After identifying the biochemical characteristics of JCK-7158, its *in vitro* antifungal activity against various phytopathogenic fungi was assessed and its agar-diffusible antifungal substances and VOCs were characterized. Moreover, its disease control efficacy against FHB in rice was assessed under greenhouse and field conditions. Furthermore, the increase in expression levels of various plant immune-related genes caused by JCK-7158 was assessed to understand the induced resistance pathway. These findings will be used as basic data for the development of a new microbial fungicide for the control of FHB in rice using the *B. velezensis* JCK-7158.

## 2 Materials and methods

### 2.1 Isolation of bacterial strains from rice

To select strains exhibiting antifungal activity against phytopathogenic fungi and having the ability to induce resistance, various rice samples were collected from rice fields at Chonnam National University and Gokseong in the Republic of Korea. The collected rice samples were categorized into leaves, stems, roots, and grains. Each rice sample (10 g) was then mixed with 100 mL of sterile distilled water and blended. After filtration through four layers of cheesecloth, the filtrates of leaves, stems, roots, and grains were diluted by 100-, 40-, 1,000-, and 100-fold, respectively (De Curtis et al., 2010). Subsequently, 100 μL of each dilution was spread onto tryptic soy agar (TSA; Becton, Dickinson and Co., Sparks, MA, United States) plates. The plates were incubated at 30°C for 1–3 days. Single colonies were isolated from TSA plates based on their morphological characters and further incubated on TSA at 30°C for 1 day. A total of 606 strains were isolated. Following this, each strain was incubated in tryptic soy broth (TSB; Becton, Dickinson and Co.) at 30°C with shaking at 150 rpm for 1 day. The strains were stored at −80°C in 30% glycerol until use.

### 2.2 Culture condition of strains and preparation of cell-free culture filtrate

The strains were first incubated on TSA at 30°C for 1 day. A single colony from each strain was then incubated in TSB at 30°C with rotary shaking at 150 rpm for 3 days. The culture broth from each bacterial strain was centrifuged at 13,000 rpm for 5 min to obtain the supernatant. The supernatants containing extracellular compounds were then filtered through a 0.2-μm membrane syringe filter to obtain axenic culture filtrates for antimicrobial activity testing (Han et al., 2022).

### 2.3 Minimum inhibitory concentration (MIC) test

A 96-well microtiter plate was used to screen antagonistic bacteria with antifungal activity based on the mycelial growth inhibitory effects of their axenic culture filtrates against two pathogenic strains: *F. graminearum* KACC 47495 and Z-3639. *F. graminearum* KACC 47495 was obtained from the Korea Agricultural Culture Collection (Jeonju, Republic of Korea), and *F. graminearum* Z-3639 was obtained from Prof. Yin-Won Lee of Seoul National University. The two pathogenic strains were incubated in potato dextrose broth (PDB) at 25°C for 5 days and filtered through Miracloth. Following this, the mycelia were washed with sterile distilled water. After weighing the mycelia, sterile distilled water was added at a concentration of 50 mg/mL and the mycelia were homogenized using a homogenizer HG-15D (DAIHAN Scientific, Kangwon, Republic of Korea) at 10,000 rpm for 5 s to prepare a mycelial suspension. Then, the mycelial suspension (1%) was added to PDB. Different concentrations of culture filtrates (0.078–10%) were obtained by performing serial twofold dilutions of the culture filtrates of potential antagonistic strains (Nguyen et al., 2019). PDB was used as a negative control. Each well in the 96-well plate received a final volume of 100 μL of the fungal suspension treated with the culture filtrates of antagonistic bacteria. The plates were incubated at 25°C for 2–3 days. The experiments were conducted in triplicate.

### 2.4 Identification of JCK-7158

JCK-7158 was selected by screening antagonistic bacteria exhibiting antifungal activity against *F. graminearum* KACC 47495 and *F. graminearum* Z-3639. The genomic DNA of JCK-7158 was extracted using the Dneasy Blood and Tissue Kit (Qiagen, Hilden, Germany), according to the manufacturer’s instructions, and the conserved region of 16S ribosomal ribonucleic acid (rRNA) was amplified by PCR (Han et al., 2022). The PCR thermal profile consisted of an initial denaturation step at 95°C for 5 min; 30 cycles of 95°C for 30 s, annealing at 55°C or 65°C for 30 s, and extension at 72°C for 90 s; and a final extension step at 72°C for 10 min. The amplified fragments were sequenced using the primer 27F/1429R at Genotech Crop (Daejeon, Republic of Korea). The sequence of JCK-7158 16S rRNA was compared for similarity with the reference *Bacillus* sequences in the GenBank database using the Basic Local Alignment Search Tool (BLAST). Phylogenetic analysis was performed on MEGA version 6 using the maximum-likelihood method with 1,000 bootstrapping trials (Han et al., 2022).

### 2.5 Biochemical characterization of JCK-7158

#### 2.5.1 Extracellular enzyme activity

The extracellular enzyme activity experiment of JCK-7158 strain was performed by applying Kirby-Bauer’s disc diffusion method using the following medium inducing extracellular enzyme production (Hudzicki, 2009): protease (1% skim milk +1.5% agar; Becton, Dickinson and Co., Franklin Lakes, NJ, United States), chitinase (1% colloidal chitin +1.5% agar; Becton, Dickinson and Co., Franklin Lakes, NJ, United States), cellulase (0.4% carboxymethyl cellulose sodium +1.5% agar; Sigma-Aldrich, St. Louis, MS, United States), gelatinase (10% gelatin +1.5% agar; Duksan, Ansan-si, Republic of Korea). Colloidal chitin for the chitinase test was prepared according to the method previously described (Hsu and Lockwood, 1975). First, 100 mL of 12 M HCl was added to crab shell powder (10 g) and stirred for 6 h. After 6 h, about 1 L of cold distilled water was added and stirred to sufficiently mix, and then centrifuged at 4°C and 4,500 rpm for 20 min to obtain a chitin precipitate. The precipitate was washed 4–5 times with distilled water, and the pH was adjusted to 7. The precipitate adjusted to pH 7 was autoclaved and stored at room temperature. A sterile paper disk was placed on each medium, and the axenic culture filtrate obtained from the 3-day-old TSB culture of JCK-7158 strain was dispensed at 5 μL, 10 μL, and 15 μL each for protease, chitinase, and cellulase tests, and 20 μL, 40 μL, and 60 μL each for gelatinase test. A sterile TSB medium was used as a negative control. All the experiments were performed in triplicates. After incubating for 1–9 days at 30°C, the clear zones were observed either directly for protease and gelatinase activities or after staining for cellulase and chitinase. Cellulase and chitinase media were stained by adding 5 mL of Lugol’s solution (2.5 g/L iodine and 5 g/L potassium iodide) to the plate for visualization.

#### 2.5.2 Plant growth hormone indole-3-acetic acid production

An experiment was performed to confirm the production of the plant growth hormone indole-3-acetic acid (IAA) in the culture filtrate of the JCK-7158 strain. JCK-7158 was incubated in 5 mL of TSB containing L-tryptophan (150 mg/L) at 30°C and 150 rpm for 3 days. The culture broth was centrifuged at 4°C and 13,000 rpm for 5 min, and the supernatant was filtered through a 0.2 μm membrane syringe filter. Then, 1 mL of the axenic culture filtrate was mixed with 2 mL of Salkowski’s reagent (150 mL H_2_SO_4_, 250 mL sterile water, 7.5 mL 0.5 M FeCl_3_·6H_2_O). After maintaining it for 20 min under room temperature and dark conditions, it was confirmed whether the mixture showed a pink color or not. TSB containing L-tryptophan (150 mg/L) was used as a negative control. The experiment was repeated twice in triplicates.

#### 2.5.3 Acetoin production

2,3-Butanediol has been known as a plant resistance inducer and its precursor is acetoin. To check the acetoin production by JCK-7158, the Voges-Proskauer (VP) method was used (Renna et al., 1993). A single colony of JCK-7158 was inoculated into 5 mL of MR-VP medium (7 g/L peptone, 5 g/L dextrose, 5 g/L dipotassium phosphate) and incubated at 30°C and 150 rpm for 3 days. Then, 100 μL of the culture broth, 60 μL of 5% 1-naphthol dissolved in ethanol, and 20 μL of 40% potassium hydroxide dissolved in distilled water were added to a 96-well plate in this order. It was maintained at room temperature for 10 to 15 min, and it was checked whether the mixed solution exhibited a cherry red color or not. A cherry red color indicated a positive result that the culture broth contains acetoin produced by a strain and a yellow–brown color indicated a negative result that the culture broth does not contain acetoin. MRS-VP medium was used as a negative control, and MR-VP medium supplemented with acetoin (1 mg/mL) was used as a positive control. The experiment was repeated twice in triplicates.

### 2.6 *In vitro* antifungal activity bioassay

To assess the mycelial growth inhibitory activity of the culture broth of JCK-7158 against various phytopathogenic fungi, a dual culture bioassay was performed. The phytopathogenic fungi used in the experiment were as follows: *Botrytis cinerea*, *Colletotrichum coccodes*, *F. asiaticum*, *F. graminearum*, *F. oxysporum* f. sp. *cucumerinum*, *F. oxysporum* f. sp. *lycopersici*, *F. verticillioides*, *Gaeumannomyces graminis*, *Phytophthora infestans*, *Pythium ultimum*, *Rhizoctonia solani* AG 2-2(IV) causing brown patch, *R. solani* AG 2-2(IV) causing large patch, and *R. solani* AG-4. The phytopathogenic fungi were cultured on PDA at 25°C for 5 days. Agar plugs with mycelia were collected with a 6-mm-diameter cork borer and placed at a point 2 cm away from the edge of the PDA plate. At 5 cm from the pathogen, each strain was streaked for a length of 5 cm. The untreated control groups were inoculated with the pathogens only. The experiments were three times repeated in triplicate. The plates were incubated at 25°C for 5 days. The inhibition rate was the mean of three runs with three replicates.

To assess the mycelial growth inhibitory activity of the culture filtrates against various phytopathogenic fungi, the following fungi were used: *B. cinerea*, *Clarireedia jacksonii*, *C. coccodes*, *F. asiaticum*, *F. graminearum*, *F. oxysporum* f. sp. *cucumerinum*, *F. oxysporum* f. sp. *lycopersici*, *F. verticillioides*, *G. graminis*, *P. infestans*, *R. solani* AG 2–2 (IV) causing large patch, and *R. solani* AG-4. The method was the same as that described for the MIC test.

### 2.7 Isolation and identification of antifungal metabolites

A 3-day-old TSB culture of JCK-7158 (3 L) was centrifuged at 4,500 rpm for 20 min to obtain the culture supernatant (2.9 L). The culture supernatant was successively extracted twice with ethyl acetate (EtOAc) and butanol (BuOH). The organic solvent extracts were evaporated using a rotary evaporator (N-1110; EYELA Co., Tokyo, Japan) to obtain 594.8 mg of EtOAc extract and 8.1483 g of BuOH extract. Then, the antifungal activity of each solvent extract against *F. graminearum* KACC 47495 was tested using the 96-well microtiter plate method.

The active antifungal substance was separated from the BuOH layer, which exhibited strong antifungal activity. An aliquot of BuOH extract dissolved in methanol at a concentration of 50 mg/mL was loaded onto a thin layer chromatography (TLC) plate (20 × 20 cm, 0.2 cm thickness; Merck, Darmstadt, Germany) and eluted with chloroform:methanol:water (14:6:1, v/v/v). The TLC plate was then sterilized using UV light and placed on PDA medium incorporated with 1% *F. graminearum* suspension. The plate was incubated at 25°C, and the mycelial growth inhibitory activity was confirmed. Then, the most active fraction was loaded onto a preparative TLC (prep-TLC) plate (20 × 20 cm, 0.5 cm thickness; Merck, Darmstadt, Germany) and developed using the same solvent system. Five regions were separated, and the silica gel powder was then scraped off. Following this, elution was performed with methanol to obtain five fractions (BF1–BF5). The antifungal activity of the five fractions was tested. The active fraction BF4 was loaded onto the prep-TLC plate and developed using the chloroform:methanol:water (55:36:8, v/v/v) solvent system. Four regions were separated, and the silica gel powder from each region was scraped off. Following this, elution was performed with methanol to obtain four fractions (BF41–BF44). The four fractions and three cyclolipopeptide (CLP) standards (iturin A, fengycin, and surfactin; Sigma-Aldrich, St. Louis, MS, United States) were analyzed by TLC using chloroform:methanol:water (55:36:8, v/v/v). The TLC plate was then developed, observed under UV light at 254 and 365 nm wavelengths, and visualized by spraying p-anisaldehyde and water. The antifungal activity of the four fractions was also tested.

The antifungal metabolites of the partially purified fraction BF4-1 were analyzed by UHPLC-Q-Orbitrap-MS. The UPLC-heated electrospray ionization (HESI)-MS system consisted of the Dionex Ultimate 3,000 UHPLC module and a HESI quadrupole Orbitrap mass spectrometer (Thermo Scientific, Bremen, Germany) controlled by Xactive Tune 1.1 and Xcalibur 2.2 software (Thermo Fisher Scientific, San Jose, USA), in addition to a C_18_ reverse-phase column (Acquity UPLC BEH C_18_ Column, 1.7 μm, 2.1 × 100 mm); it was maintained at 40°C. The mobile phases consisted of solvent A (0.1% formic acid in H_2_O) and solvent B (0.1% formic acid in acetonitrile). The flow rate of the two solvents was 300 μL/min, and the injection volume was 2 μL. Gradient elution was performed as follows: 0–10 min, 95% A and 5% B; 10–15 min, 100% B; and 16–20 min, 95% A and 5% B. The conditions of the HESI quadrupole Orbitrap mass spectrometer were as follows: resolution, 70,000; collision energy, 30 V.

### 2.8 Assessment of antifungal activity of VOCs

To assess the antifungal activity of VOCs produced by JCK-7158, 14 phytopathogenic fungi were used: *B. cinerea*, *Clarireedia jacsonii*, *C. coccodes*, *F. asiaticum*, *F. graminearum*, *F. oxysporum f*. sp. cucumerinum, *F. oxysporum* f. sp. *lycopersici*, *F. verticillioides*, *G. graminis*, *P. infestans*, *P. ultimum*, *R. solani* AG 2-2(IV) causing brown patch, *R. solani* AG 2-2(IV) causing large patch, and *R. solani* AG-4. The phytopathogenic fungi were cultured on PDA at 25°C for 3–7 days. To block direct contact, a bi-Petri dish with a separate center was used. PDA medium was dispensed on one side of the plate, and TSA medium was dispensed on the other side (Lim et al., 2017). Agar mycelium plugs (6 mm diameter) containing fresh mycelia of the phytopathogens from 3–7 days culture were cut using a sterile cork borer (6 mm diameter) and were inoculated at the center of the PDA medium. The JCK-7158 strain was streaked on the other side. The untreated samples were inoculated with pathogens only, without bacterial inoculum. The experiment was conducted in triplicate. The plates were sealed twice with Parafilm to prevent volatile organic compounds from leaking and incubated at 25°C. Fungal growth was assessed based on the diameter of mycelial growth, and the inhibition rate was calculated as a percentage (%). The experiments were three times repeated in triplicate and the inhibition rate was the mean of three runs with three replicates.

### 2.9 GC–MS analysis of VOCs produced by JCK-7158

To analyze VOCs produced by the JCK-7158 strain, solid phase microextraction (SPME) GC–MS analysis was performed. The JCK-7158 strain was inoculated into 5 mL of TSB and precultured at 30°C with shaking at 150 rpm for 1 day. An aliquot of the preculture was inoculated into 20 mL of TSB at a concentration of 1% and incubated at 30°C with shaking at 150 rpm for 3 days. VOCs produced by JCK-7158 were collected using SPME fiber (Supelco, Bellefonte, PA, USA) in the headspace at 50°C for 30 min (Xie et al., 2018). The samples were analyzed by GC–MS using Shimadzu GC–MS QP2010 (Shimadzu, Kyoto, Japan) equipped with a DB-5 capillary column (30 m × 0.25 mm i.d. × 0.25 μm film thickness, Agilent). Helium was used as the mobile phase, and the flow rate was maintained at 1.0 mL/min. The injector temperature was maintained at 250°C. The column temperature was maintained at 60°C for 2 min, then increased to 250°C at a rate of 10°C/min, and maintained at 250°C for 20 min. The ionization voltage of the mass spectrometer was 70 eV, and the analysis was performed in the positive ion mode with a scan range of 50–400 m/z at 200°C.

The obtained mass spectrum was identified by comparing it with the data of the WILEY8 Library, and the content of each material was expressed as the area ratio of the total ion chromatogram (TIC) peak. The experiment was conducted in triplicate and uninoculated TSB medium was used as a negative control.

### 2.10 Formulation of JCK-7158

To assess the disease control activity of JCK-7158 against FHB in rice, wettable powder (WP) and suspension concentrate (SC) formulations of JCK-7158 culture broths were prepared. Oxidized starch (Floset Light; Daesang, Seoul, Republic of Korea) was added to a 3-day-old TSB culture broth of JCK-7158 at a concentration of 20% and then spray dried. The spray drying conditions were as follows: inlet temperature, 140°C; air blower, 10 m^3^/h; deblock, 50; and pump, 10 rpm. WP and SC formulations were prepared using the spray-dried material of JCK-7158 culture broth. The composition of the WP formulation was as follows: spray-dried material of JCK-7158, 10 g; white carbon, 15 g; CR-SDS, 12.5 g; CR-WP100, 12.5 g; and kaolin, 50 g. Each component was added and thoroughly mixed. The composition of the SC formulation was as follows: spray-dried material of JCK-7158, 100 g; CR-NF135B, 30 mL; propylene glycol, 50 mL; xanthan gum 1.0% solution, 100 mL; sodium benzoate, 2 g; CR-SAG672, 1 mL; and distilled water, 719 mL. Each component was added and stirred for 3 h.

### 2.11 Histochemical β-glucuronidase (GUS) assay using *Arabidopsis* leaves

Transgenic *A. thaliana* plants expressing GUS fused with the pathogenesis-related gene 1 (*PR1*) promoter were used to examine the expression of *PR1*, a known induced resistance marker gene. The seeds were initially sterilized with 95% ethanol for 30 s and then sterilized with a bleach solution (2% NaOCl, 0.05% Tween 20) for 5 min. The remaining solution was washed thrice with sterile distilled water and stored for 2 days at 4°C under dark conditions (Mishra et al., 2016). The immersed seeds were placed on half-strength Murashige–Skoog (MS) medium (2.2 g MS salt, 10 g sucrose, and 8 g phyto agar/L) containing 50 μg/mL kanamycin (Deng et al., 2021). The seeds were incubated on MS medium for 12 days with a photoperiod of 16 h in a plant growth chamber at 25°C and 80% relative humidity. Various concentrations of culture broth, culture filtrate, and cell suspension of the JCK-7158 strain and two JCK-7158 formulations (SC and WP) were used as samples. The JCK-7158 strain was incubated at 30°C with shaking at 150 rpm for 1 day. All treatment groups were diluted with sterile distilled water by 500-, 1,000-, 2,000-, and 4,000-fold, respectively, following which 2.5 mL of each culture diluent was poured into each well of a 24-well plate. Two 12-day-old *A. thaliana* plants were placed in each well and maintained at room temperature in an orbital shaker for 48 h. SA (0.1 and 0.01 mM) was used as a positive control, and TSB (0.1%) was used as a negative control. GUS staining was performed using a previously reported method (Kondo et al., 2014). After 48 h, the plants were immersed in 90% acetone at 4°C for 1 h to fix reacted plants and then washed twice with 0.1 M sodium phosphate buffer (pH 7.0), followed by immersion in GUS staining solution [0.1% Triton-X, 2.5 mM ethylenediaminetetraacetic acid, 100 mM sodium phosphate buffer, 2.5 mM potassium ferrocyanide, 2.5 mM potassium ferricyanide, and 2 mM X-GlucA (Duchefa, X1405)] under dark conditions at 4°C. The plates were incubated overnight in a 30°C water bath. The staining solution was then removed and the reaction mixture was fixed in 70% ethanol for 1 h, followed by immersion in 90% ethanol at 1-h intervals to remove chlorophyll. The blue color was observed using a microscope (Stemi 508; Carl Zeiss, Germany).

### 2.12 *In vivo* bioassay

Rice seeds (*Oryza sativa* cv. Samkwang) provided by the National Institute of Crop Science (Rural Development Administration, Suwon, Republic of Korea) were used for the rice FHB bioassay. The preparation method of rice plants is described in detail in the Supplementary Information.

A sample was prepared with a 3-day-old TSB culture broth of JCK-7158 (OD_600_ = 0.8). The 3-day-old TSB culture broth of JCK-7158 was then centrifuged at 10,000 rpm for 10 min, and the supernatant was filtered through a 0.2-μm membrane syringe filter to obtain the culture filtrate. An equal amount of distilled water was added to the pellet to obtain the cell suspension. The culture broth, culture filtrate, and cell suspension of JCK-7158 were further diluted with distilled water by 1,000- and 2,000-fold, following which Tween 20 was added at a concentration of 250 μg/mL. Both SC and WP formulations of JCK-7158 were treated at 1,000- and 2,000-fold dilutions, respectively. Before applying the culture broth, culture filtrate, and cell suspension samples, the rice plants were pretreated with Tween 20 solution (250 μg/mL). The JCK-7158 samples were applied twice by foliar spray at the booting and heading stages at a 1-week interval. Distilled water containing 250 μg/mL of Tween 20 was used as a negative control for the culture broth, culture filtrate, and cell suspension samples. Distilled water was used as a negative control for the formulations. The synthetic fungicide Peulrei (13% propiconazole, 13% difenoconazole; Syngenta Co., Seoul, Republic of Korea) was used as a positive control and was treated at a 2,000-fold dilution a day before inoculation.

The treated rice plants were inoculated with the rice FHB pathogen *F. asiaticum* 031 at the flowering stage a week after the second application of the JCK-7158 samples. *F. asiaticum* 031 was isolated from the infected tissue of rice by Prof. Seo Jeong-Ah (Soongsil University, Seoul, Republic of Korea). The pathogen was cultured on PDA at 25°C for 4–5 days. Then, agar plugs containing the mycelia of *F. asiaticum* 031 were placed in carboxymethyl cellulose broth and incubated at 25°C with shaking at 150 rpm under light conditions for 7 days. The carboxymethyl cellulose broth of *F. asiaticum* 031 was collected through filtration with four layers of Miracloth to obtain spores (Strange and Smith, 1971). The spore suspension was counted under a microscope (Axio Imager.A2; Carl Zeiss, Yena, Germany) using a hemocytometer. The density of the spore suspension was adjusted to 2.0 × 10^5^ spores/mL, and Tween 20 was then added to the spore suspension at a concentration of 500 μg/mL. The pathogen was inoculated onto rice plants by spraying 1.6 mL spore suspension on each rice panicle. Disease severity was visually evaluated 7 days after pathogen inoculation, with the percentage ranging from 0 to 100%. The experiments were repeated three times in triplicate; each replicate consisted of six panicles. Disease severity, disease incidence, FHB index, and control value of FHB index were calculated using Equations 1–4, respectively. The values were the mean ± standard deviations of three runs with three replicates each.


(1) $$\begin{array}{l} \mathrm{Disease}\;\mathrm{severity}\;\left(\%\right)=\\ \mathrm{percentage}\;\mathrm{of}\;\mathrm{infected}\;\mathrm{grains}\;\mathrm{among}\;\mathrm{the}\;\mathrm{treated}\;\mathrm{grains}\; \end{array}$$

(2) $$\begin{array}{l} \mathrm{Disease}\;\mathrm{incidence}\;\left(\%\right)=\\ \mathrm{percentage}\;\mathrm{of}\;\mathrm{infected}\;\mathrm{spikes}\;\mathrm{among}\;\mathrm{the}\;\mathrm{treated}\;\mathrm{spikes}\; \end{array}$$

(3) $$FHB\;\mathrm{index}=\left(\mathrm{Disease}\;\mathrm{severity}\times \mathrm{Disease}\;\mathrm{incidence}\right)/100$$

(4) $$\begin{array}{l} \mathrm{Control}\;\mathrm{value}\;\mathrm{of}\;FHB\;\mathrm{index}\;\left(\%\right)\\ =\left(FHB\;\mathrm{index}\;\mathrm{of}\;\mathrm{control}-\frac{FHB\;\mathrm{index}\;\mathrm{of}\;\mathrm{treatment}}{FHB\;\mathrm{index}\;\mathrm{of}\;\mathrm{control}}\right)\times 100\; \end{array}$$

### 2.13 Field test

The control efficacy of JCK-7158 SC against FHB in rice was evaluated in a rice field (*O. sativa* cv. Samkwang) located at Chonnam National University, Gwangju, Republic of Korea. The replicate plots of the field experiment were 1 × 1 m plots, with a spacing of 1 m between plots. The experiment was conducted in triplicate and using a randomized complete block design. A total of 90 spikes (30 spikes per replicate) were used per treatment. JCK-7158 SC was diluted by 1,000- and 2,000-fold with water. Each sample, approximately 1 L per plot, was treated by foliar spray 2 weeks (booting stage) and 1 week (heading stage) before inoculation. The treatment concentrations and timings were based on the results of *in vivo* bioassay in this study and our previous paper (Jeon et al., 2022). Distilled water was used as a negative control, and the synthetic fungicide Peulrei was used as a positive control, which was applied a day before pathogen inoculation. *F. asiaticum* 031 was prepared and inoculated into the rice plants, as described in the *in vivo* bioassay. Disease severity and disease incidence were assessed thrice at 2-week intervals from 2 weeks after pathogen inoculation. The formulae used to calculate disease severity, disease incidence, and control values are described in the previous section.

### 2.14 Mycotoxin analysis

Mycotoxin analysis was conducted to determine whether the accumulation of *Fusarium* mycotoxins, such as NIV and ZEA, in rice grains is reduced on treatment with JCK-7158 SC under the field condition. The grains were harvested 6 weeks after pathogen inoculation and dried for 3 days, following which the dried samples were coarsely ground.

NIV was extracted using a DON-NIV WB column (Vicam, Milford, USA), according to the manufacturer’s recommendations. The extracts were completely dried using nitrogen gas and subsequently redissolved in 1 mL of sterile distilled water:acetonitrile:methanol (90:5:5, v/v/v) solution. The resulting solution was then filtered through a 0.22-μm membrane filter. The experiment was conducted in triplicate. The mycotoxin was quantitively analyzed using the Waters ACQUITY UPLC H-Class System equipped with a PDA detector at 218 nm. The column used was Xselect CSH C_18_ (2.1 × 100 mm, 2.5 μm); it was maintained at 40°C. The mobile phases consisted of solvent A (distilled water), solvent B (acetonitrile), and solvent C (methanol) (90:5:5, v/v/v). The flow rate was 0.3 mL/min for 10 min, and the injected volume was 10 μL.

ZEA was extracted using a ZearalaTest™ WB column (Vicam, Milford, USA), according to the manufacturer’s recommendations. The extracts were eluted with 1.5 mL of methanol. The methanol solution was mixed with 1.5 mL of sterile distilled water and then filtered through a 0.22-μm membrane filter. The samples were analyzed using UPLC-PDA (Waters, Scotland) in triplicate. The mycotoxin was quantitively analyzed using the Waters ACQUITY UPLC H-Class System equipped with an FLD detector at λ_ex_ 274 nm and λ_em_ 440 nm. The column used was Xselect CSH C_18_ (2.1 × 100 mm, 2.5 μm); it was maintained at 40°C. The mobile phases consisted of solvent A (distilled water), solvent B (acetonitrile), and solvent C (methanol) (43:35:22, v/v/v). The flow rate was 0.3 mL/min for 10 min, and the injected volume was 10 μL.

### 2.15 RNA extraction and quantitative real-time PCR analysis of defense-related genes

In rice, the effects of JCK-7158 on the expression of defense-related genes were analyzed to understand the defense mechanism of JCK-7158 against FHB. The culture broth, culture filtrate, and cell suspension of JCK-7158 (1,000-fold dilution) were treated by foliar spray 2 weeks and 1 week before inoculation into rice. Following this, the treated rice samples were inoculated with *F. asiaticum* spores (2.0 × 10^5^ spore/mL) in Tween 20 solution (250 μg/mL). Tween 20 solution (250 μg/mL) was used as a negative control. Rice leaves were collected 1, 3, and 7 days after the first treatment; 1, 3, and 7 days after the second treatment; and 1 and 2 days after inoculation of the pathogen. The total RNA of rice leaves was extracted using the IQeasy™ Plus Plant RNA Extraction Mini Kit (iNtRON Biotechnology, Seong-nam, Republic of Korea), according to the manufacturer’s recommendations. cDNA was synthesized using the oligo-(dT) primer and SuperScript™ IV reverse transcriptase (Invitrogen Inc., Carlsbad, CA, United States), according to the manufacturer’s instructions. Real-time PCR was performed on a real-time PCR detection system (Bio-Rad CFX 96; Bio-Rad Laboratories, Hercules, CA, United States) using iQ™ SYBR Green Supermix (BioRad Laboratories), according to the manufacturer specifications.

The PCR primers used in this study were synthesized by Genotech (Daejeon, Republic of Korea) and are listed in Supplementary Table S1. The actin gene was used as a reference gene (Jannoey et al., 2017). The phenylalanine ammonia-lyase gene (*OsPAL1*), rice nonexpressor of PR gene 1 (*OsNPR1*), *PR1*, and *PR10* were used as SA signaling pathway-related genes (Sathe et al., 2019; Cheng et al., 2020). The lipoxygenase gene (*LOX*) was involved in the JA signaling pathway (Sathe et al., 2019), and the 1-aminocyclopropane-1-carboxylic acid synthase gene (*OsACS1*) was involved in the ET signaling pathway (Yoo et al., 2018). The catalase gene (*CAT*) and alternative oxidase gene (*AOX1a*) were recognized as genes related to reactive oxygen species (ROS) scavenging (Jannoey et al., 2017; He et al., 2021).

### 2.16 Statistical analysis

All statistical data were expressed as the mean ± standard deviation (SD) of replicates using SPSS software (version 21.0 for Windows; SPSS, IBM Corp., Armonk, NY, United States). Statistical differences were assessed using one-way ANOVA and determined using Duncan’s test (*p* < 0.05).

## 3 Results

### 3.1 Strain selection using MIC test

Of the 606 bacterial strains, 10 strains exhibited strong antifungal activity against *F. graminearum* KACC 47495 and *F. graminearum* Z-3639 (Table 1). Of these 10 strains, the JCK-7158 strain exhibited the strongest antifungal activity with an MIC value of 1.25%. Therefore, the JCK-7158 strain was further studied.

**Table 1:** *In vitro* antifungal activity of the 10 bacterial strains against *Fusarium graminearum*.

JCK strain no.	MIC value (%)	
	*F. graminearum* KACC 47495	*F. graminearum* Z-3639
7004	5	2.5
7098	5	5
7120	5	5
7144	10	10
7157	2.5	2.5
**7158**	**1.25**	**1.25**
7159	10	10
7443	5	5
7540	10	10
7563	5	5

The bold value showed the lowest MIC value against *Fusarium graminearum* and *Fusarium asiaticum*.

### 3.2 Identification of JCK-7158

**Figure 1:**
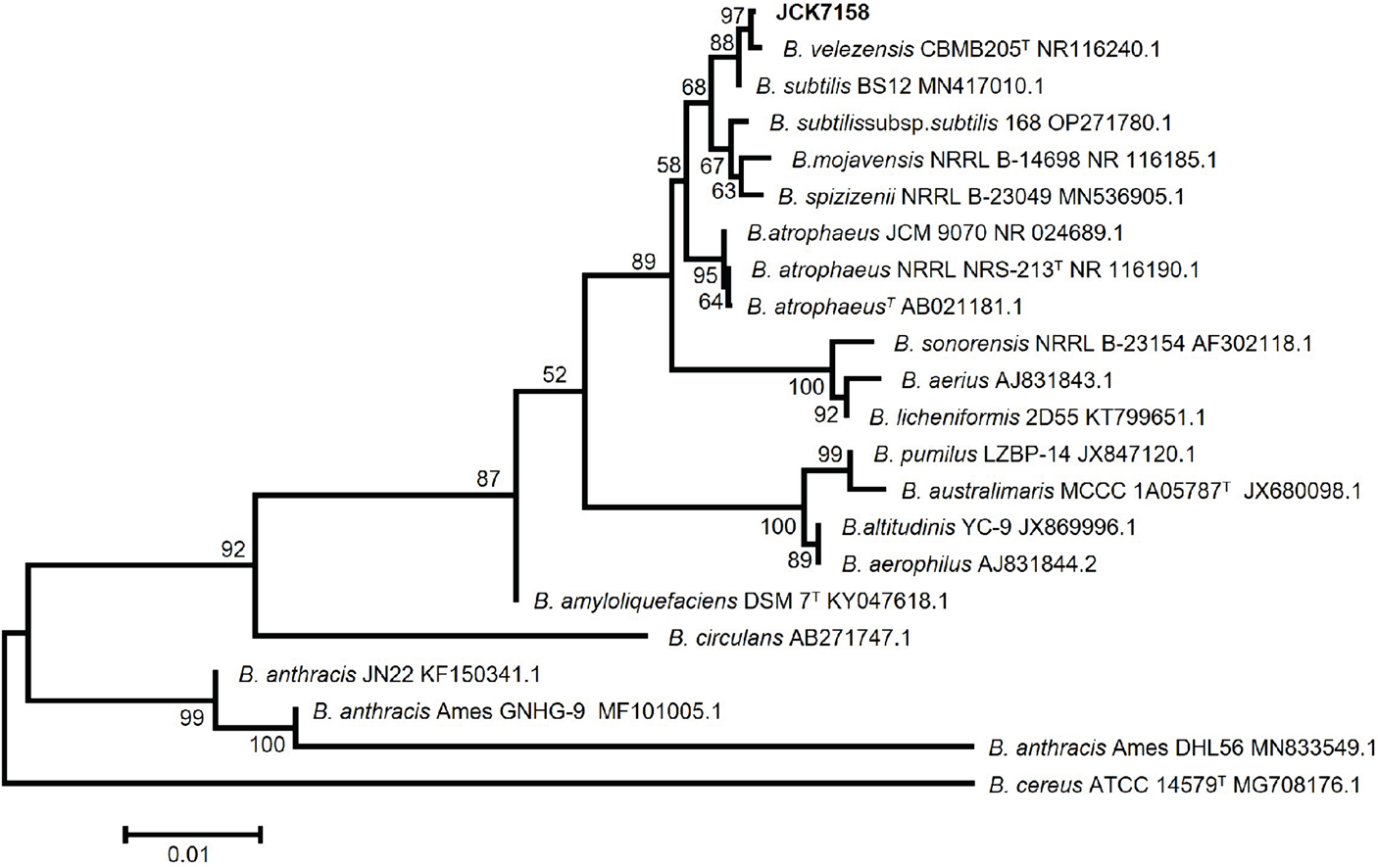
Neighbor-joining phylogenetic tree based on Kimura’s two-parameter + G model using the concatenated nucleotide sequences of 16S rRNA representing the species in the genus *Bacillus.* Bootstrap support values above 60% (from 1,000 replicates) are given at the nodes. The scale bar represents substitution sites.

Based on phylogenetic analysis (Figure 1) and BLAST analysis against the NCBI database using the 16S rRNA sequence of JCK-7158, the strain was identified as *B. velezensis*. This strain was deposited in the Korean Collection for Type Cultures (KCTC) as KATC15169BP.

### 3.3 Biochemical characterization of JCK-7158

JCK-7158 produced extracellular enzymes, such as protease, chitinase, cellulase, and gelatinase (Supplementary Figure S1). Secondary metabolites, including the 2,3-butanediol precursor acetoin and the plant growth hormone IAA, were also released by JCK-7158 (Supplementary Figure S2).

### 3.4 *In vitro* antifungal activity of JCK-7158

The MIC test was performed to examine the *in vitro* antifungal activity of JCK-7158 against various phytopathogenic bacteria. The JCK-7158 strain exhibited the strongest antifungal activity with MIC values of 1.25% against *C. jacksonii* and *F. graminearum.* It also exhibited antifungal activity against *B. cinerea*, *C. coccodes*, and *G. graminis*, among others, with MIC values of 5%. These results confirmed that the JCK-7158 strain exhibited strong antifungal activity against various phytopathogenic fungi.

A dual culture bioassay was also performed to assess the mycelial growth inhibitory activity of JCK-7158 against various phytopathogenic fungi. The JCK-7158 strain exhibited more than 50% inhibitory activity against most pathogens used in the experiment. In particular, it exhibited strong mycelial growth inhibitory activity against *B. cinerea*, *F. oxysporum* f. sp. *cucumerinum*, and *F. asiaticum* (Table 2; Supplementary Figure S3).

**Table 2:** Antifungal activity of the JCK-7158 strain against phytopathogenic fungi based on the minimum inhibitory concentration (MIC) test and dual culture bioassay.

Phytopathogenic fungi	MIC value (%)	Inhibition rate (%)^*^
*Botrytis cinerea*	5	59.91 ± 0.94
*Clarireedia jacksonii*	1.25	-
*Colletotrichum coccodes*	5	50.03 ± 4.75
*Fusarium asiaticum*	10	56.20 ± 1.80
*Fusarium graminearum*	1.25	51.55 ± 2.20
*Fusarium oxysporum* f. sp. *cucumerinum*	-	57.98 ± 2.48
*Fusarium oxysporum* f. sp. *lycopersici*	-	53.13 ± 3.94
*Fusarium verticillioides*	-	53.84 ± 3.57
*Gaeumannomyces graminis*	5	51.37 ± 0.72
*Phytophthora infestans*	5	52.08 ± 1.18
*Pythium ultimum*	-	19.10 ± 3.82
*Rhizoctonia solani* AG 2–2 (IV) causing brown patch	-	47.28 ± 1.31
*Rhizoctonia solani* AG 2–2 (IV) causing large patch	5	56.12 ± 1.53
*Rhizoctonia solani* AG-4	5	46.04 ± 0.66

^*^Mycelial growth inhibition rate (%) = (1 − the length of the mycelial growth in the treatment/the length of the mycelial growth in the control) × 100. Each value represents the mean ± standard deviation with three replicates.

### 3.5 Isolation and identification of antifungal metabolites

**Figure 2:**
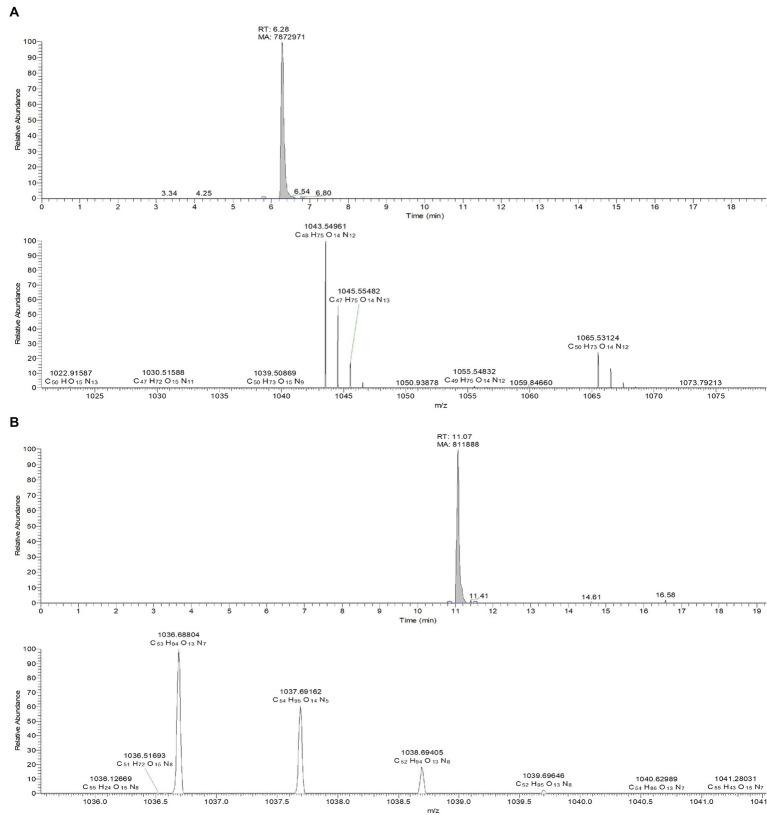
LC-TOF-ESI-MS analysis of the bioactive compounds from the active fraction BF4-1. **(A)** Iturin A. **(B)** Surfactin.

The culture filtrate of JCK-7158 was extracted with EtOAc and BuOH to isolate the antifungal metabolites. The BuOH extract of JCK-7158 exhibited antifungal activity against *F. graminearum* KACC 47495, as assessed by bioautography (Supplementary Figure S4A). The BuOH extract of JCK-7158 showed an MIC value of 125 μg/mL against *F. graminearum* KACC 47495. Among the five fractions obtained from prep-TLC, the BF4 fraction was the most active against *F. graminearum* KACC 47495. To isolate pure substances from the BF4 fraction, prep-TLC was performed and four fractions were obtained. Of these, BF4-1, BF4-2, and BF4-3 were found to contain iturin A as a major substance (Supplementary Figure S4B). BF4-1 exhibited antifungal activity against *F. graminearum* KACC 47495 with an MIC value of 125 μg/mL. LC–MS analysis of the BF4-1 fraction revealed an active metabolite, iturin A, with a molecular weight of 1043.5495 in the positive ion mode. Moreover, the chemical composition of another active compound having a peak molecular weight of 1036.68757 in the positive ion mode was C_53_H_93_O_13_N_7_, which was consistent with that of surfactin (Ma et al., 2016; Figure 2). *In vitro,* the antifungal activity of BF4-1 was assessed against various phytopathogenic fungi using the MIC method. BF4-1 exhibited the lowest MIC value of 31.25 μg/mL against *C. coccodes*, followed by *C. jacksonii*, *R. solani* AG 2–2 (IV) causing large patch, and *R. solani* AG-4 (Supplementary Table S2).

### 3.6 Antifungal activity of VOCs

JCK-7158 not only produced antifungal compounds but also generated VOCs that inhibited the growth of various phytopathogenic fungi (Supplementary Figure S5). The inhibition efficacy against *B. cinerea* was the strongest at approximately 51%, followed by *F. graminearum* at approximately 49% (Table 3).

**Table 3:** Antifungal activity of volatile organic compounds produced by JCK-7158 against phytopathogenic fungi.

Phytopathogenic fungi	Inhibition rate (%)^*^
*Botrytis cinerea*	60.51 ± 8.86
*Clarireedia jacksonii*	12.56 ± 5.63
*Colletotrichum coccodes*	27.57 ± 4.85
*Fusarium asiaticum*	21.05 ± 13.58
*Fusarium graminearum*	49.20 ± 18.01
*Fusarium oxysporum* f. sp. *cucumerinum*	29.49 ± 2.13
*Fusarium oxysporum* f. sp. *lycopersici*	7.26 ± 1.11
*Fusarium verticillioides*	28.97 ± 12.36
*Gaeumannomyces graminis*	21.30 ± 9.26
*Phytophthora infestans*	30.00 ± 5.93
*Pythium ultimum*	8.94 ± 3.47
*Rhizoctonia solani* AG 2–2 (IV) causing brown patch	31.46 ± 6.88
*Rhizoctonia solani* AG 2–2 (IV) causing large patch	28.81 ± 7.34
*Rhizoctonia solani* AG-4	9.39 ± 1.56

For colony area measurement, colony area (mm^2^) = length of colony (mm) × width of colony (mm). ^*^Inhibition rate (%) = [(Colony area of the control group in mm^2^ − Colony area of the treatment group in mm^2^)/(Colony area of the control group in mm^2^)] × 100. Each value represents the mean ± standard deviation of three runs, with three replicates each.

### 3.7 Analysis of VOCs using GC–MS

VOCs produced by JCK-7158 inhibited the growth of various phytopathogenic fungi. Therefore, GC–MS analysis was performed to identify VOCs produced by JCK-7158. Five VOCs were detected within 7 min (Supplementary Table S3). By comparing the mass spectra of the substances in the library, the chemical structures of the five substances were found to be 6-methylheptan-2-one (34.89%), heptane-2-one (24.06%), 5-methyl-2-heptanone (20.75%), 2,5-dimethylpyrazine (15.94%), and 5-methylhexan-2-one (4.36%). Of these, the JCK-7158 strain produced 6-methylheptan-2-one as a major volatile substance.

### 3.8 Qualitative induction of *PR1* expression

**Figure 3:**
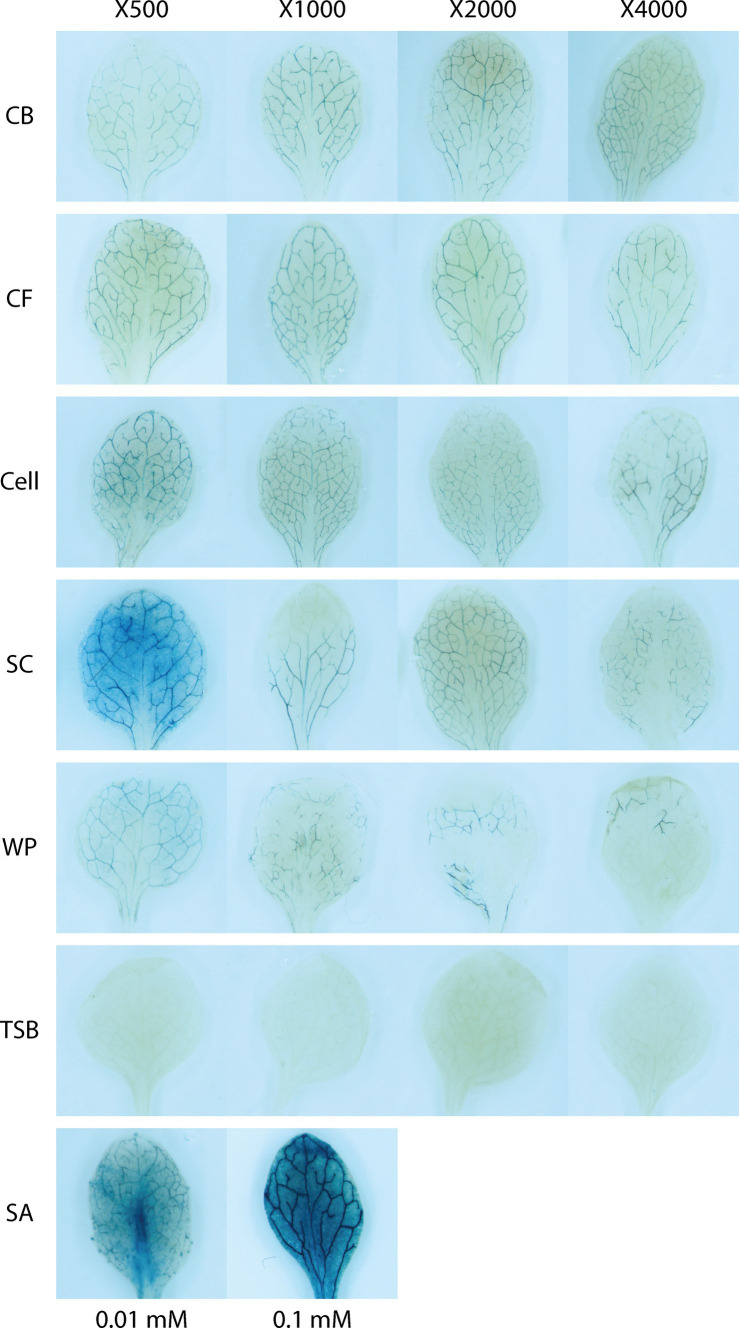
GUS expression induced by JCK-7158 in *PR1::GUS* in *Arabidopsis* plant seedlings.

All JCK-7158 treatments, such as the culture broth, culture filtrate, cell suspension, and SC and WP formulations, showed positive reactions with regard to *PR1::GUS* expression. As shown in Figure 3, a blue color appeared along the leaf veins in transgenic *A. thaliana* leaves in all treatments. However, the untreated control did not show any blue color. The positive control SA also showed a very strong blue color in the treated leaves.

### 3.9 Disease control efficacy of JCK-7158 against FHB in rice under greenhouse conditions

**Figure 4:**
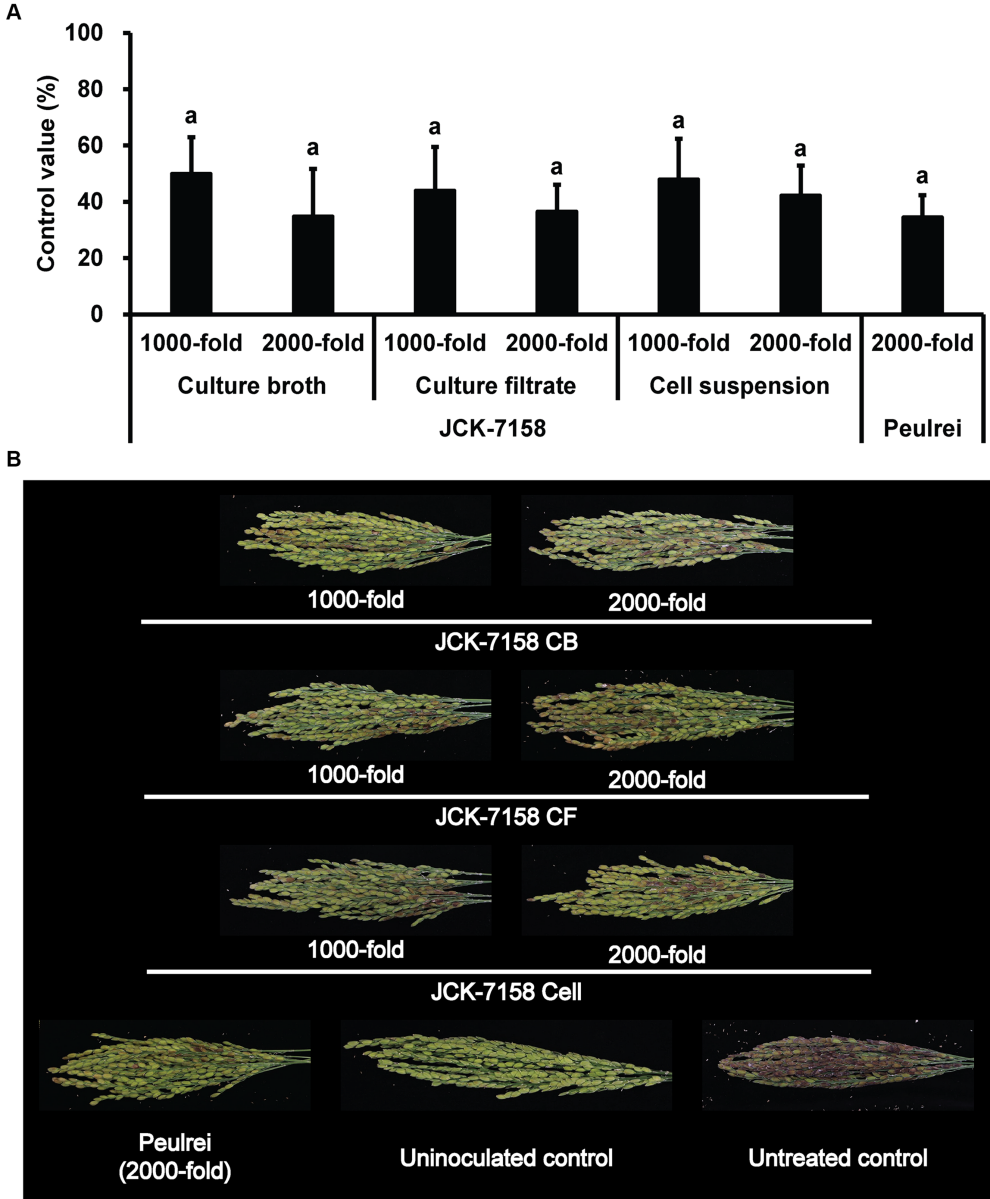
Disease control efficacy of JCK-7158 against *Fusarium* head blight in rice. **(A)** Mean percentage control value of JCK-7158 and Peulrei against *Fusarium* head blight in rice 7 days after inoculation. **(B)** Symptom of *Fusarium* head blight in rice. Each value represents the mean ± standard deviation of three runs, three replicates (pots; six panicles per pot) each. Lowercase letters indicate that the value is not significantly different from other values with the same letter at the *p* < 0.05 level, according to Duncan’s test.

**Figure 5:**
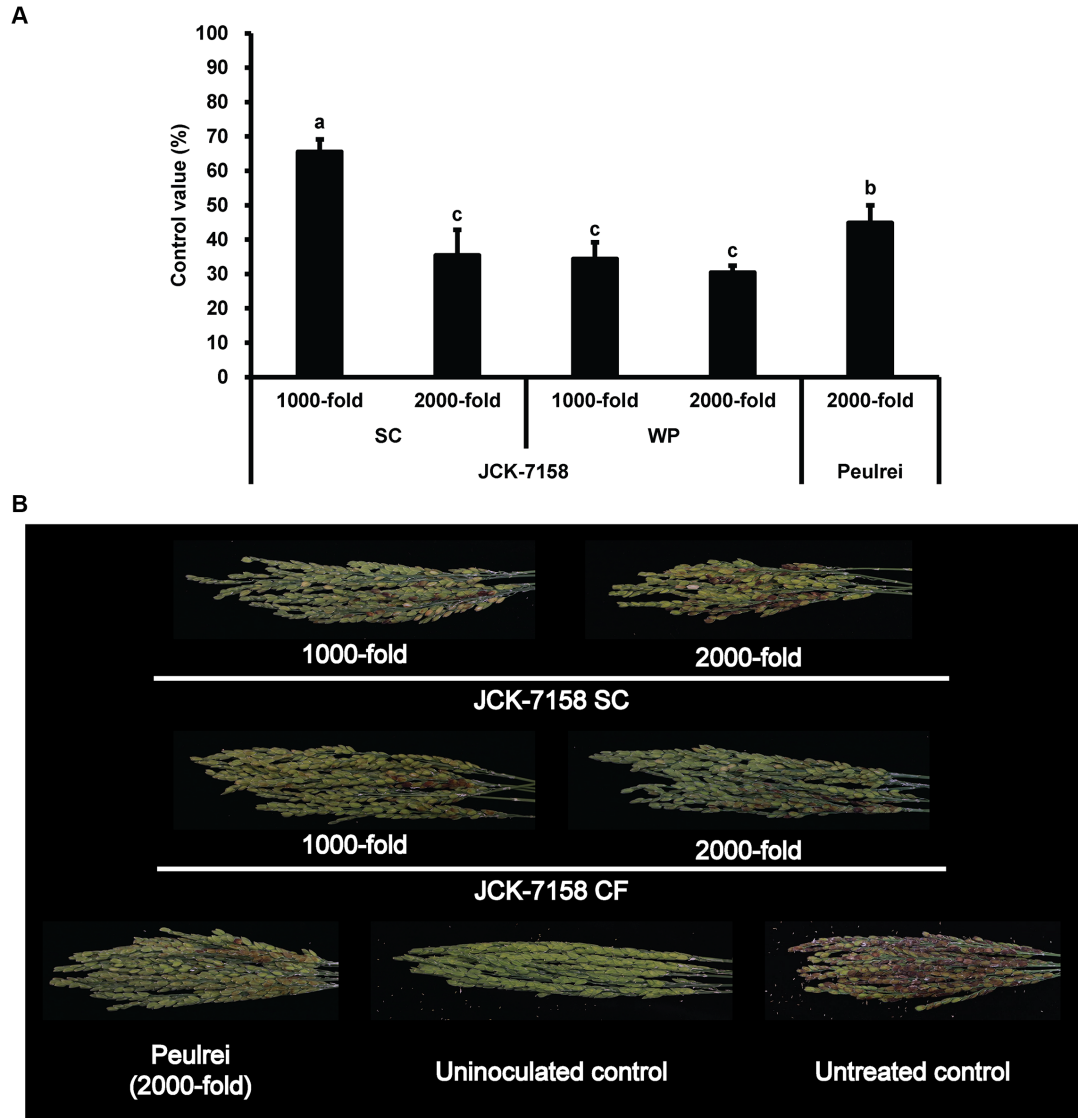
Disease control efficacy of JCK-7158 formulations against *Fusarium* head blight in rice. **(A)** Mean percentage control value of JCK-7158 formulations and Peulrei against *Fusarium* head blight in rice 7 days after inoculation. **(B)** Symptom of *Fusarium* head blight in rice. Each value represents the mean ± standard deviation of three runs, three replicates (pots; six panicles per pot) each. Lowercase letters indicate that the value is not significantly different from other values with the same letter at the *p* < 0.05 level, according to Duncan’s test.

The culture broth, culture filtrate, and cell suspension of JCK-7158 effectively controlled the development of FHB in rice (Table 4; Figure 4). At 1,000- and 2,000-fold dilutions, the disease control values were 50 and 35% for the culture broth, 44 and 36% for the culture filtrate, and 48 and 42% for the cell suspension, respectively, which were similar to the findings in the positive control group. Meanwhile, out of the two JCK-7158 formulations (WP and SC) under greenhouse conditions, the 1,000-fold diluted SC formulation most effectively reduced the development of FHB in rice with a control value of 65.5%; this value was higher than that in the positive control group (44.9%) (Table 5; Figure 5). The SC formulation exhibited higher disease control efficacy than the WP formulation.

**Table 4:** Disease control efficacy of the culture broth, culture filtrate, and cell suspension of JCK-7158 against *Fusarium* head blight in rice under greenhouse conditions.

Treatment	Dilution rate	Disease severity (%)	Disease incidence (%)	FHB index^*^	Control value of FHB index (%)
JCK-7158 culture broth	1,000-fold	47.33 ± 13.05^a^	100.00 ± 0.00^a^	47.33 ± 13.05^a^	49.88 ± 13.82^a^
	2,000-fold	61.67 ± 17.01^a^	100.00 ± 0.00^a^	61.67 ± 17.01^a^	34.71 ± 18.01^a^
JCK-7158 culture filtrate	1,000-fold	53.00 ± 15.62^a^	100.00 ± 0.00^a^	53.00 ± 15.62^a^	43.88 ± 16.54^a^
	2,000-fold	60.00 ± 9.54^a^	100.00 ± 0.00^a^	60.00 ± 9.54^a^	36.47 ± 10.10^a^
JCK-7158 cell suspension	1,000-fold	49.20 ± 14.47^a^	100.00 ± 0.00^a^	49.20 ± 14.47^a^	47.91 ± 15.32^a^
	2,000-fold	54.61 ± 10.74^a^	100.00 ± 0.00^a^	54.61 ± 10.74^a^	42.18 ± 11.38^a^
Peulrei^**^	2,000-fold	61.94 ± 7.88^a^	100.00 ± 0.00^a^	61.94 ± 7.88^a^	34.41 ± 8.34^a^
Untreated control	-	94.44 ± 4.74^b^	100.00 ± 0.00^a^	94.44 ± 4.74^b^	0.00 ± 5.02

^*^FHB index = (Disease severity × Disease incidence)/100. ^**^Peulrei was used as a positive control. Each value represents the mean ± standard deviation of three runs, three replicates (pots; six panicles per pot) each. Lowercase letters indicate that values are not significantly different from other values with the same letter at the *p* < 0.05 level, according to Duncan’s test.

**Table 5:** Disease control efficacy of SC and WP formulations of JCK-7158 against *Fusarium* head blight in rice under greenhouse conditions.

Treatment	Dilution rate	Disease severity (%)	Disease incidence (%)	FHB index^*^	Control value of FHB index (%)
JCK-7158 SC	1,000-fold	32.78 ± 4.29^a^	95.83 ± 7.22^a^	31.28 ± 3.24^a^	65.53 ± 3.57^a^
	2,000-fold	58.58 ± 6.71^c^	100.00 ± 0.00^a^	58.58 ± 6.71^c^	35.43 ± 7.40^c^
JCK-7158 WP	1,000-fold	59.51 ± 4.38^c^	100.00 ± 0.00^a^	59.51 ± 4.38^c^	34.40 ± 4.83^c^
	2,000-fold	63.13 ± 1.82^c^	100.00 ± 0.00^a^	63.13 ± 1.82^c^	30.42 ± 2.00^c^
Peulrei^**^	2,000-fold	50.01 ± 4.63^b^	100.00 ± 0.00^a^	50.01 ± 4.63^b^	44.87 ± 5.10^b^
Untreated control	-	90.72 ± 3.23^d^	100.00 ± 0.00^a^	90.72 ± 3.23^d^	0.00 ± 3.56

^*^FHB index = (Disease severity × Disease incidence)/100. ^**^Peulrei was used as a positive control. Each value represents the mean ± standard deviation of three runs, three replicates (pots; six panicles per pot) each. Lowercase letters indicate that values are not significantly different from other values with the same letter at the *p* < 0.05 level, according to Duncan’s test.

### 3.10 Disease control efficacy of JCK-7158 SC against FHB in rice under field conditions

**Figure 6:**
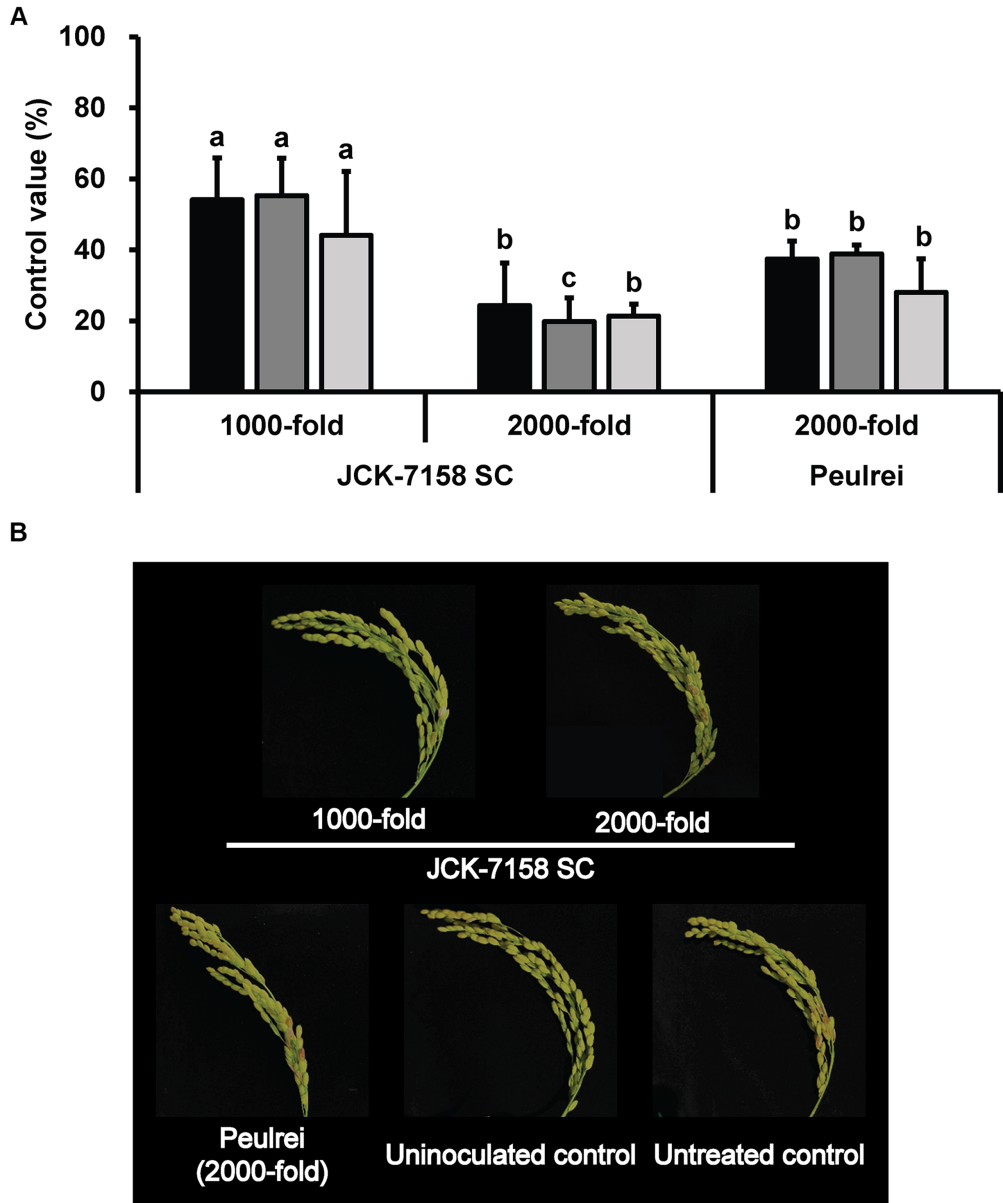
Disease control efficacy of the SC formulation of JCK-7158 against *Fusarium* head blight in rice under field conditions. **(A)** Mean percentage control value of JCK-7158 SC and Peulrei against *Fusarium* head blight in rice at 2, 4, and 6 weeks after inoculation. **(B)** Symptom of *Fusarium* head blight in rice. Each value represents the mean ± standard deviation of three plots (30 panicles per plot). Lowercase letters indicate that the value is not significantly different from other values with the same letter at the *p* < 0.05 level, according to Duncan’s test.

To assess the disease control efficacy of JCK-7158 SC under field conditions, 1,000- and 2,000-fold dilutions of JCK-7158 SC were applied 2 weeks and 1 week before pathogen inoculation. At 2, 4, and 6 weeks after pathogen inoculation, treatment with 1,000-fold diluted JCK-7158 SC exhibited control values of 54, 55, and 44%, respectively, while treatment with 2,000-fold diluted JCK-7158 SC exhibited control values of 24, 20, and 21%, respectively. The control values of the positive control Peulrei were 37, 39, and 28% after 2, 4, and 6 weeks, respectively. Thus, the 1,000-fold diluted JCK-7158 SC formulation controlled FHB in rice more effectively than the synthetic fungicide Peulrei (Table 6; Figure 6).

**Table 6:** Disease control efficacy of JCK-7158 SC against *Fusarium* head blight in rice under field conditions.

Treatment		JCK-7158 SC		Peulrei^***^	Uninoculated control	Untreated control
		1,000-fold	2,000-fold	2,000-fold		
Disease severity	2 WAI^**^	2.59 ± 0.50^b^	3.97 ± 0.49^c^	3.37 ± 0.19^bc^	1.04 ± 0.30^a^	5.00 ± 0.80^d^
	4 WAI	2.53 ± 0.41^b^	4.20 ± 0.33^d^	3.30 ± 0.22^c^	0.87 ± 0.20^a^	5.09 ± 0.64^e^
	6 WAI	4.85 ± 1.53^ab^	6.86 ± 0.40^cd^	6.21 ± 0.82^bc^	3.36 ± 0.43^a^	8.62 ± 1.34^d^
Disease incidence (%)	2 WAI	87.78 ± 6.74^b^	95.00 ± 3.33^cd^	92.78 ± 2.55^bc^	50.00 ± 1.67^a^	100.00 ± 0.00^d^
	4 WAI	89.44 ± 6.74^b^	97.22 ± 2.55^bc^	94.44 ± 2.55^bc^	59.44 ± 5.09^a^	100.00 ± 0.00^c^
	6 WAI	99.44 ± 0.96^b^	98.89 ± 1.92^b^	100.00 ± 0.00^b^	94.44 ± 4.19^a^	100.00 ± 0.00^b^
FHB index^*^	2 WAI	2.29 ± 0.59^b^	3.78 ± 0.60^c^	3.13 ± 0.25^bc^	0.52 ± 0.16^a^	5.00 ± 0.80^d^
	4 WAI	2.28 ± 0.54^b^	4.08 ± 0.34^d^	3.11 ± 0.13^c^	0.52 ± 0.14^a^	5.09 ± 0.64^e^
	6 WAI	4.83 ± 1.55^ab^	6.78 ± 0.29^cd^	6.21 ± 0.82^bc^	3.19 ± 0.54^a^	8.62 ± 1.34^d^
Control value of FHB index (%)	2 WAI	54.10 ± 11.76^a^	24.32 ± 11.96^b^	37.38 ± 5.10^b^	-	0.00
	4 WAI	55.21 ± 10.54^a^	19.78 ± 6.75^c^	38.88 ± 2.54^b^	-	0.00
	6 WAI	44.03 ± 18.01^a^	21.39 ± 3.32^b^	28.00 ± 9.45^b^	-	0.00

^*^FHB index = (Disease severity × Disease incidence)/100. ^**^Weeks after inoculation. ^***^Peulrei was used as a positive control. Each value represents the mean ± standard deviation of three plots (thirty panicles per plot). Lowercase letters indicate that values are not significantly different from other values with the same letter at the *p* < 0.05 level, according to Duncan’s test.

### 3.11 Effect of JCK-7158 SC treatment on rice yield

Rice yield was measured by weighing 30 spikes with husk. Compared with the untreated control, the weight of rice grains increased on treatment with JCK-7158 SC at a 1,000-fold dilution. The yield of the untreated control rice was 97.47 g, while that of rice treated with 1,000-fold diluted JCK-7158 SC was 107.91 g. Thus, 1,000-fold diluted JCK-7185 SC significantly increased rice yield compared with the untreated control (Table 7; Supplementary Figure S6).

**Table 7:** Effect of JCK-7158 SC on rice yield in plants with *Fusarium* head blight under field conditions.

Treatment	Dilution rate	Total weight (g)^*^
JCK-7158 SC	1,000-fold	107.91 ± 5.61^ab^
	2,000-fold	98.32 ± 7.74^bc^
Peulrei^**^	2,000-fold	113.93 ± 4.63^a^
Uninoculated control	-	114.83 ± 2.76^a^
Untreated control	-	97.47 ± 5.09^c^

^*^Weight of 30 panicles. ^**^Peulrei was used as a positive control. Each value represents the mean ± standard deviation of three plots (30 panicles per plot). Lowercase letters indicate that the value is not significantly different from other values with the same letter at the *p* < 0.05 level, according to Duncan’s test.

### 3.12 Mycotoxin analysis

Mycotoxins, such as NIV and ZEA, were quantitatively analyzed in the rice grains treated with JCK-7158 SC. Only NIV was detected in all the samples. The NIV content of rice treated with 1,000-fold diluted JCK-7158 SC was 169.29 μg/kg, while that of the untreated control rice was 285.09 μg/kg (Table 8), indicating a 40.62% decrease in NIV accumulation in rice grains on treatment with 1,000-fold diluted JCK-7158 SC.

**Table 8:** Effect of JCK-7158 and Peulrei fungicide on the contamination of nivalenol (NIV), deoxynivalenol (DON), and zearalenone (ZEA) under rice field conditions.

Treatment	Dilution rate	NIV content (μg/kg)	Reduction of NIV content (%)	DON content (μg/kg)	ZEA content (μg/kg)
JCK-7158 SC	1,000-fold	169.29 ± 17.73^a^	40.62 ± 6.22^a^	ND	ND
	2,000-fold	343.59 ± 96.50^b^	0.00 ± 33.85^b^	ND	ND
Peulrei^*^	2,000-fold	224.89 ± 5.24^ab^	21.12 ± 1.84^ab^	ND	ND
Uninoculated control		ND	-	ND	ND
Untreated control		285.09 ± 73.19^ab^	0.00 ± 25.67^ab^	ND	ND

^*^Peulrei was used as a positive control. Means with the same letters are not significantly different (*p* < 0.05), according to Tukey’s test. ND means not detected. Each value represents the mean ± standard deviation of three repeated values from two trials.

### 3.13 Expression of defense-related genes using qRT-PCR

**Figure 7:**
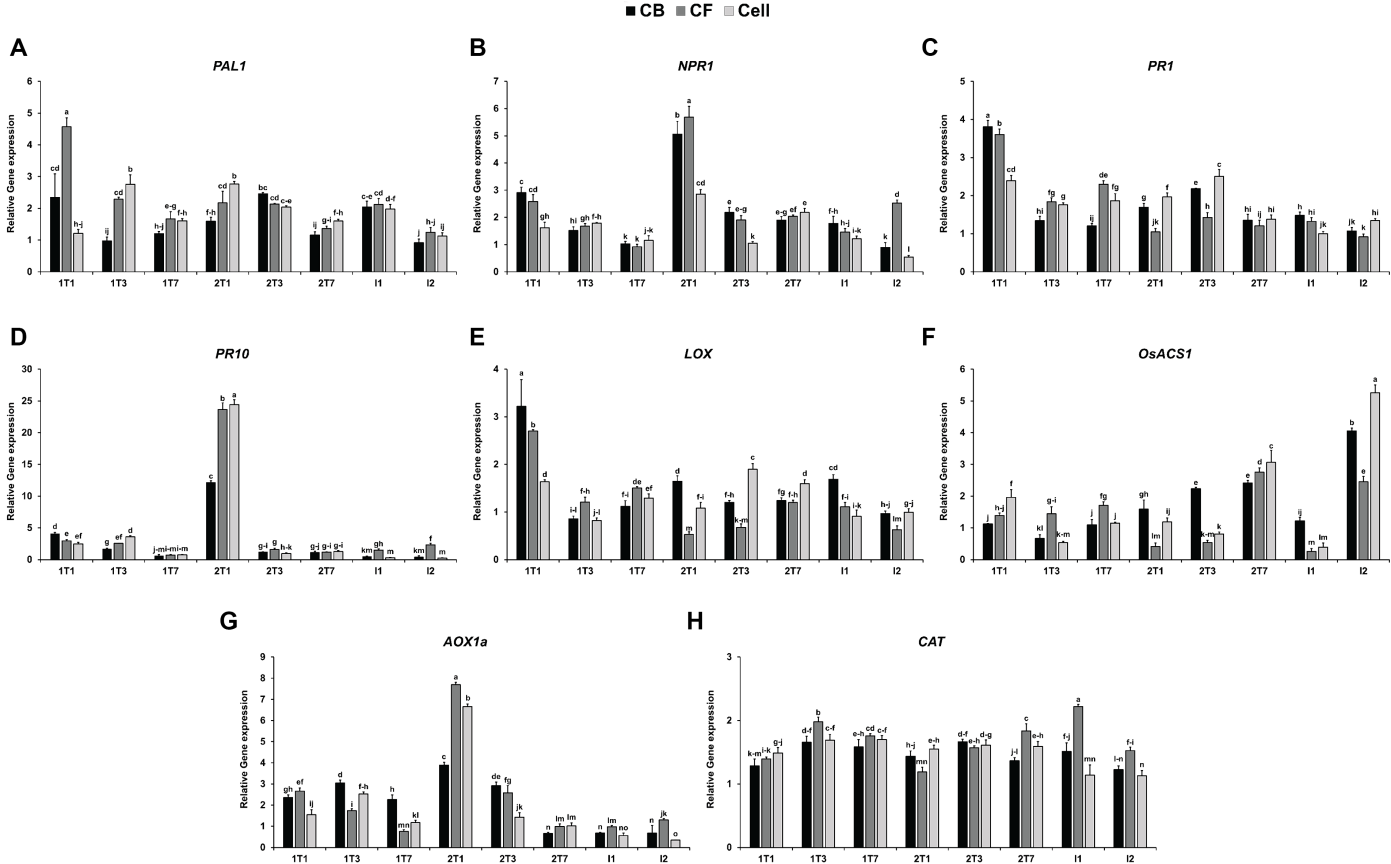
Induction of rice defense-related gene expression by the culture broth, culture filtrate, and cell suspension of JCK-7158 at 1, 3, and 7 days after treatment and 1 and 2 days after inoculation. Expression levels of defense genes, namely the SA signaling pathway-related genes **(A)** *PAL1*, **(B)** *NPR1*, **(C)** *PR1*, and **(D)** *PR10*; the JA signaling pathway-related gene **(E)** *LOX*; the ET-related gene **(F)** *OsACS1*; and the ROS signaling pathway-related genes **(G)** *AOX1* and **(H)** *CAT*, in rice plants were quantified. The data are expressed as the mean ± standard deviation. Lowercase letters indicate that values are not significantly different from other values with the same letter at the *p* < 0.05 level, according to Duncan’s test.

To assess the effect of JCK-7158 on the expression of plant defense-related genes, qRT-PCR was performed using JCK-7158-treated rice leaves. Compared with the untreated control group, the expression level of *PAL1* was upregulated by 2.3- and 4.57-fold in leaves treated with the culture broth and culture filtrate of JCK-7158, respectively, in the 1 T1 group (1 day after the first treatment). In cell suspension-treated leaves, the expression level of *PAL1* was significantly upregulated in the 1 T3 group (3 days after the first treatment) and the 2 T1 group (1 day after the second treatment) (Figure 7A). Compared with the untreated control group, the expression level of *NPR1* was upregulated by 5.06-, 5.68- and 2.85-fold on treatment with the culture broth, culture filtrate, and cell suspension of JCK-7158, respectively, in the 2 T1 group (Figure 7B). Moreover, compared with the untreated control group, the expression level of *PR1* was upregulated by 3.81-, 3.60-, and 2.40-fold on treatment with the culture broth, culture filtrate, and cell suspension of JCK-7158, respectively, in the 1 T1 group (Figure 7C). Compared with the untreated control group, the expression level of *PR10* was also upregulated by 12.16-, 23.65-, and 24.43-fold on treatment with the culture broth, culture filtrate, and cell suspension of JCK-7158, respectively, in the 2 T1 group (Figure 7D).

A gene involved in the JA-related pathway, *LOX*, was upregulated by 3.22- and 2.70-fold in leaves treated with the culture broth and culture filtrate of JCK-7158, respectively, in the 1 T1 group. Moreover, *LOX* expression was significantly upregulated in leaves treated with the cell suspension of JCK-7158 in the 2 T3 group (Figure 7E). A gene involved in ET-related pathways, *OsACS1*, was significantly upregulated following all treatments in the 2 T7 and I2 groups (Figure 7F). Of the genes involved in ROS-related pathways, *AOX1* was significantly upregulated by 2.36-, 2.66-, and 1.54-fold on treatment with the culture broth, culture filtrate, and cell suspension of JCK-7158, respectively, in the 2 T1 group (Figure 7G). *CAT* was upregulated following all treatments in 1 T1–I1 groups (Figure 7H). Thus, JCK-7158 enhanced the expression of all of the SA, JA, and ET signaling pathway-related genes and ROS-scavenging genes tested in this study.

## 4 Discussion

Although many studies have been conducted worldwide on the biocontrol of FHB in barley, wheat, and corn, among others, few studies have assessed the biocontrol of FHB in rice. Therefore, we searched for a new biocontrol agent that could control FHB in rice.

In this study, of the 606 strains isolated from rice, the JCK-7158 strain was selected because it exhibited excellent antifungal and induced resistance activities. The JCK-7158 strain was identified as *B. velezensis* based on phylogenetic analysis using the 16S rRNA sequence. This antagonistic strain produced the plant growth hormone IAA, acetoin, and various extracellular enzymes (such as protease, chitinase, gelatinase, and cellulase). *Bacillus velezensis* secretes plant hormones, such as the auxin IAA, to promote the growth of plant hair roots and roots, improve the absorption of nutrients in plants, and increase plant growth and yield by promoting cell division and differentiation (Hong et al., 2022). Moreover, acetoin, a precursor of 2,3-butanediol, is a metabolite produced by various microorganisms, such as *B. subtilis*, *Lactococcus lactis*, *Leuconostoc mesenteroides*, and *Hanseniaspora guilliermondii* (Xu et al., 2011). 2,3-Butanediol is known to induce plant defense mechanisms that encourage plants to defend themselves against disease and promote plant growth (Magno-Perez-Bryan et al., 2015). Various extracellular enzymes, such as endochitinase, chitobiosidase, and glucanase, secreted by beneficial microorganisms exhibit antifungal activity by inhibiting the germination of phytopathogenic fungal spores and by inhibiting their mycelial growth (Lorito et al., 1994; Yuan and Crawford, 1995; Flores et al., 1997). Therefore, we predicted that JCK-7158 would potentially exhibit antifungal activity against various phytopathogenic fungi and aimed to isolate and identify compounds with antifungal activity.

For this purpose, we performed the MIC test and dual culture bioassays. The JCK-7158 strain exhibited broad-spectrum *in vitro* antifungal activity against various phytopathogenic fungi, including *F. graminearum*. Through bioassay-guided fractionation, the antifungal metabolites were identified as iturin A and surfactin. These compounds are CLPs produced by the genus *Bacillus*. These CLPs share a common structure consisting of a lipid tail linked to a short cyclic oligopeptide, and each has a different amino acid sequence (Ongena and Jacques, 2008). Iturin A not only exhibits direct antifungal activity but also inhibits biofilm formation by pathogens and induces an overall defense response in plants (Bais et al., 2004). Surfactin is a powerful biosurfactant with little antifungal activity; however, it can enhance the antifungal effect of iturin A (Tendulkar et al., 2007; Kim et al., 2010). According to a recent study, *B. velezensis* LM2303 can directly control FHB through antifungal metabolites, such as iturin A and surfactin (Chen et al., 2018). Kim et al. (2017) also found that although fengycin or surfactin alone does not exhibit inhibitory activity against spore germination in *F. graminearum*, a mixture of iturin A, fengycin, and surfactin exhibits a remarkable synergistic inhibitory effect. Furthermore, the coapplication of *B. amyloliquefaciens* JCK-12 producing iturin A, fengycin, and surfactin with chemical fungicides could exhibit synergistic *in vitro* antifungal effects and significant disease control efficacy against FHB under greenhouse and field conditions.

In addition, we confirmed that VOCs produced by JCK-7158 inhibit the growth of phytopathogenic fungi. The JCK-7158 strain produced 5-methylhexan-2-one, heptan-2-one, 2,5-dimethylpyrazine, 6-methylheptan-2-one, and 5-methyl-2-heptanone. Of these, 6-methylheptan-2-one (34.89%) was produced as a major VOC. Heptan-2-one, 6-methylheptan-2-one, and 5-methyl-2-heptanone inhibit the growth of *B. cinerea*, *Alternaria brassicicola*, *A. brassicae*, and *Sclerotinia sclerotiorum* (Lee et al., 2017). Heptan-2-one inhibits radial colony growth and spore germination in phytopathogenic fungi, such as *P. irregulare*, *R. solani*, and *F. oxysporum* (Cole et al., 1975). Moreover, 2,5-dimethylpyrazine is effective in suppressing anthrax in mangoes and enhancing their physiologically active contents, such as firmness, respiratory rate, and ET emission rate (Janamatti et al., 2022). Therefore, JCK-7158, which produces iturin A, surfactin, and various VOCs, could be used as a biocontrol agent against FHB not only in rice but also in barley and wheat.

However, various low-molecular-weight compounds, such as CLPs and 2,3-butanediol, are also known as plant resistance inducers. The JCK-7158 strain produces acetoin, a precursor of 2,3-butanediol, and two CLPs of iturin A and surfactin. The application of 2,3-butanediol induced plant defense in *Capsicum annuum* against several pathogens (Yi et al., 2016; Kong et al., 2018). Moreover, 2,3-butanediol treatment activated ISR against foliar diseases caused by *Microdochium nivale*, *R. solani*, and *S. homoeocarpa* in *Agrostis stolonifera* (creeping bentgrass) (Cortes-Barco et al., 2010). Shi et al. (2018) reported that the application of 2,3-butanediol to creeping bentgrass activated ISR against *R. solani* by inducing phytohormone and antioxidant responses. Moreover, Jiang et al. (2021) found that iturin A retarded the soft rot of cherry tomatoes caused by *Rhizopus stolonifer* by inducing both antifungal and plant resistance. Yamamoto et al. (2015) also revealed that CLPs of iturin A and surfactin produced by *B. amyloliquefaciens* S13-3 induced plant defense response in strawberries against *C. gloeosporioides.* Thus, low-molecular-weight compounds produced by JCK-7158 are associated with resistance induction in plants.

Therefore, we assessed the expression of *PR1*, a SAR-related marker gene that induces plant resistance against various pathogens. Treatment with JCK-7158 induced the expression of *PR1* related to a defense mechanism by inducing the expression of *PR1::GUS* in transgenic *Arabidopsis* plants. A previous study also found that *PR1::GUS* was expressed on treatment with *B. amyloliquefaciens* (Park et al., 2001). This suggests that many *Bacillus* strains can induce the expression of *PR1*. In this study, the culture broth, culture filtrate, and cell suspension of JCK-7158 induced a positive reaction in the *GUS* bioassay at a very low concentration (1,000-fold dilution). These samples also effectively controlled the development of FHB in rice. This indicates that the main mechanism of action of JCK-7158 for the control of FHB in rice is induced resistance rather than direct antifungal activity. Considering the quantity of iturin A and surfactin in the culture broth, the JCK-7158 strain cannot control the development of FHB in rice through direct antifungal activity. However, as the JCK-7158 strain was isolated as an endophyte from healthy rice tissue, other mechanisms of action, such as competition and antibiotic production in grains, may have been involved.

Our findings indicate that several materials are responsible for induced resistance because both culture filtrate and cell suspension samples expressed *PR1::GUS* and effectively controlled the development of FHB in rice. Various compounds, such as 2,3-butanediol, iturin A, and surfactin, may be responsible for induced resistance following JCK-7158 treatment. However, further studies are warranted to identify plant resistance inducers from the culture filtrate and cell suspension of the JCK-7158 strain.

In this study, qRT-PCR was performed to analyze whether JCK-7158 affects the expression of defense-related genes in rice. Compared with the untreated control group, the expression of *PAL1*, *NPR1*, *PR1*, and *PR10* was significantly upregulated on treatment with the culture broth, culture filtrate, and cell suspension of JCK-7158. *PAL1*, *NPR1*, *PR1*, and *PR10* are generally used as marker genes related to the SA signaling pathway (Sathe et al., 2019; Cheng et al., 2020). Moreover, compared with the untreated control group, the expression of *LOX*, *OsACS1*, *AOX1a*, and *CAT* was significantly upregulated on treatment with the culture broth, culture filtrate, and cell suspension of JCK-7158. *LOX* is a marker gene related to the JA signaling pathway (Shi et al., 2018), and *OsACS1* is a marker gene related to the ET signaling pathway (Yoo et al., 2018). *AOX1a* and *CAT* are genes related to ROS-scavenging enzymes (Jannoey et al., 2017; He et al., 2021). Therefore, JCK-7158 upregulated SA, JA, and ET signaling pathway-related genes and ROS-scavenging genes. Similarly, in a recent study, treatment with *B. velezensis* F21 upregulated SA-, JA-, and ROS-related genes to induce resistance to *Fusarium* wilt in watermelon (Jiang et al., 2021).

In Korea, *F. asiaticum* is mainly isolated from barley, rice, and wheat with FHB symptoms at a frequency of 95% (Jang et al., 2019). Therefore, when testing the *in vivo* disease control efficacy of JCK-7158 against FHB in rice, *F. asiaticum* was used as a pathogen in this study. The application of a 1,000-fold diluted JCK-7158 SC formulation effectively reduced the development of FHB in rice and the accumulation of NIV in grains. This indicates that JCK-7158 can be used as a biocontrol agent against FHB in rice. Several studies have reported that some antagonistic bacteria can control the development of FHB in wheat or barley and reduce the accumulation of trichothecenes (Baffoni et al., 2015; Kim et al., 2017; Degrassi and Carpentieri-Pipolo, 2020; Palazzini et al., 2022). Palazzini et al. (2022) reported that a combination of *B. velezensis* RC218 and chitosan effectively controlled FHB in bread and durum wheat. *B. amyloliquefaciens* FLN13 was also found to be a promising agent for reducing FHB in wheat (Baffoni et al., 2015). Moreover, *B. amyloliquefaciens* JCK-12 reduced the disease incidence of FHB in wheat (Kim et al., 2017). However, to the best of our knowledge, this study is the first to report the biocontrol of FHB in rice by the induced resistance activity of beneficial bacteria.

Taken together, *B. velezensis* JCK-7158 was selected as a new biocontrol agent from rice-derived 606 bacterial strains because it showed strong both antifungal and induced resistance activities against FHB pathogen and FHB, respectively. The strain produced iturin A and surfactin as agar-diffusible antifungal metabolites and antifungal VOCs. It also upregulated various plant defense-related genes and controlled effectively FHB and mycotoxin accumulation under greenhouse and field conditions by action mechanism of induced resistance rather than antifungal activity. Our findings indicate that JCK-7158 can reduce the development of FHB in rice and the accumulation of *Fusarium* mycotoxin by induced resistance activity. Therefore, the application of JCK-7158 may be highly effective in controlling both FHB and *Fusarium* mycotoxins. Synthetic fungicides can only be applied before the flowering stage. In comparison, the biological agent JCK-7158 has the advantage of not having limitations regarding the application time. Thus, JCK-7158 has promising potential for controlling FHB in rice and reducing *Fusarium* mycotoxin levels.

## 5 Conclusion

In this study, we found that JCK-7158 is an effective strain for the biocontrol of FHB in rice. JCK-7158 produces various active antifungal substances; however, its actual disease control efficacy is attributed to the induction of plant systemic resistance. This is the first study on the disease control efficacy of *B. velezensis* against FHB in rice. These results indicate that JCK-7158 has the potential to serve as a new biocontrol agent for the management of FHB in rice plants. Further studies on the isolation and identification of induced resistance substances, development of optimum fermentation and formulation, toxicity, and field evaluation are necessary to develop a new microbial pesticide for the control of rice FHB. In addition, for the effective control of rice FHB and mycotoxin accumulation, the control efficacy by alternative application of chemical fungicide and JCK-7158 formulation or their mixture should be evaluated under both greenhouse and field conditions.

## Data availability statement

The original contributions presented in the study are included in the article/Supplementary material, further inquiries can be directed to the corresponding author.

## Author contributions

YY: Writing – original draft, Writing – review & editing. AP: Writing – original draft, Writing – review & editing. BV: Writing – original draft. J-CK: Writing – original draft, Writing – review & editing.
