# Supplementary material for: Biocontrol of Fusarium head blight in rice using Bacillus velezensis JCK-7158
Source: Front Microbiol. 2024 Jun 10;15:1358689. doi: 10.3389/fmicb.2024.1358689 (PMC11194345; doi:10.3389/fmicb.2024.1358689)
Supplement: Supplementary file 1 [file Data_Sheet_1.DOCX]

Supplementary Information

Yu Jeong Yeo^1^, Ae Ran Park^1,2^, Bien Sy Vuong^1^, and Jin-Cheol Kim^1,2*^

^1^Department of Agricultural Chemistry, Institute of Environmentally Friendly Agriculture, College of Agriculture and Life Science, Chonnam National University, 77 Yongbong-Ro, Gwangju 61186, Republic of Korea

^2^Plant Healthcare Research Institute, JAN153 Biotech Incorporated, Gwangju 61186, Republic of Korea

*** Correspondence:**Jin-Cheol Kim, kjinc@jnu.ac.kr

Keywords: *Fusarium* head blight, *Bacillus velezensis,* induced resistance, antifungal activity, biocontrol agent.

# Supplementary Methods

## Preparation of rice plant for *in vivo* bioassay

Rice seeds (*Oryza sativa* cv. Samkwang; National Institute of Crop Science, Rural Development Administration, Suwon, Republic of Korea) were used for the rice FHB bioassay. The seeds were sterilized with a 2,000-fold dilution of the synthetic fungicide, Spotak (25% prochloraz EC; Kyungnong Co., Ltd., Seoul, Republic of Korea) for one day and then soaked in water for two days under dark conditions. These treated seeds were sown in small plastic pots filled with 80% rice nursery soil (Punong, Gyeongju, Republic of Korea). The plants were cultivated in a plant growth room at a temperature of 30±5°C for 4 weeks, maintaining a light/dark cycle of 16 h/8 h. After 4 weeks, the seedlings were transplanted into the center of the Wagner pot filled with 70% paddy soil mixed with the chemical fertilizer, Heulgsalang 21 (21% N, 6% H_3_PO_4_, 8% K, 1% MgO, 0.1% B; Namhae Chemical, Yeosu, Republic of Korea). The rice plants were grown in a greenhouse with a minimum temperature of 15-20℃ and a maximum of 30-35℃. At 20 days after transplanting, a chemical fertilizer named Super Alali (46% N; Namhae Chemical, Yeosu, Republic of Korea) was applied for the second fertilization. Two months after transplanting, NK fertilizer (24% N, 16% H_3_PO_4_, 1% MgO, 0.1% B; Farm Hannong Co., Ltd., Seoul, Republic of Korea) was used for the third fertilization.

# Supplementary Figures

**
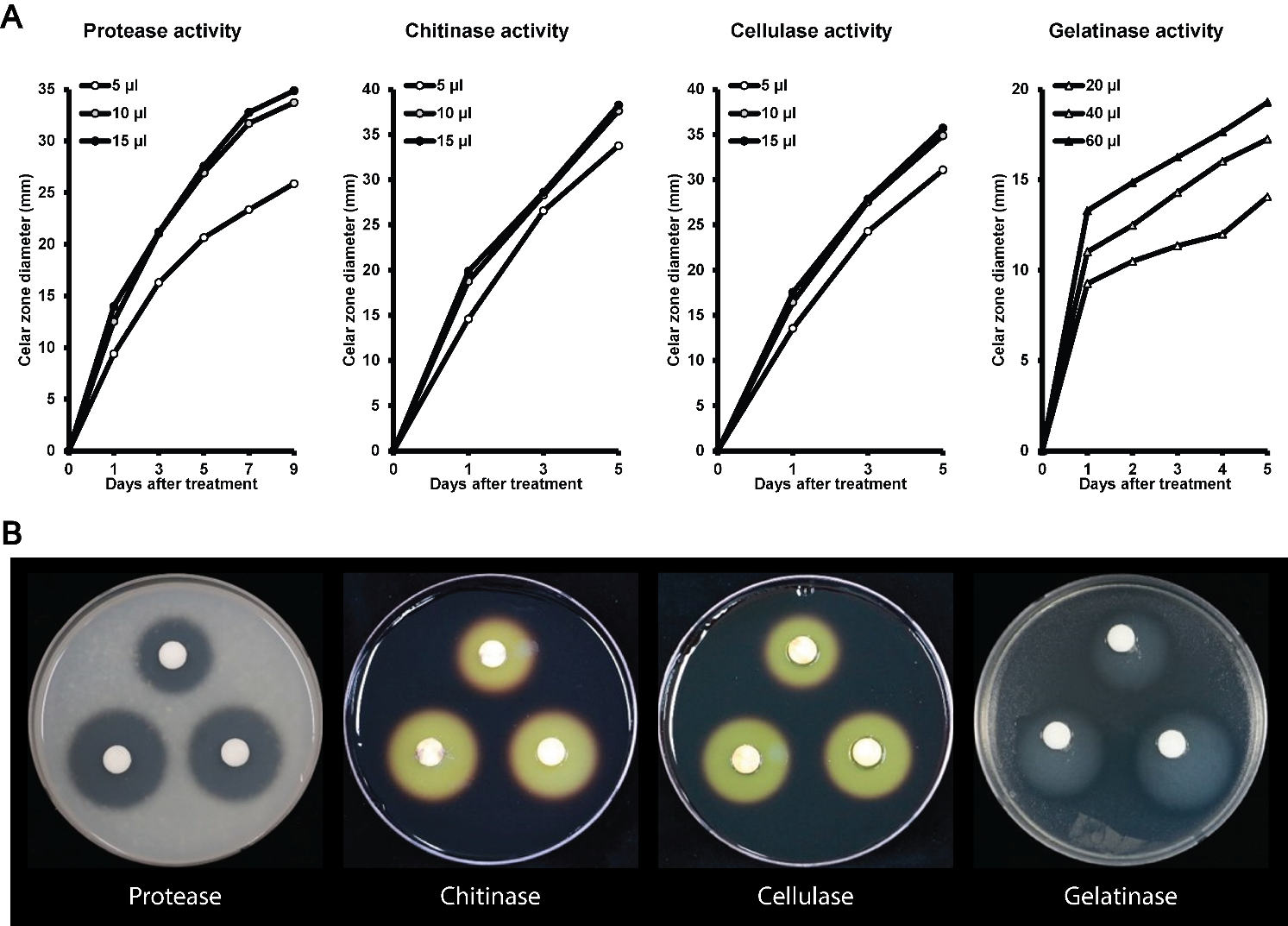
**

**Supplementary Figure 1.** Activities of extracellular enzymes produced by *Bacillus velezensis* JCK-7158. (A) Qualitative analysis results of extracellular enzymes based on the clear zone diameter of JCK-7158 culture filtrate. (B) Extracellular activities of JCK-7158 culture filtrate by the Kirby-Bauer disk diffusion method.

**
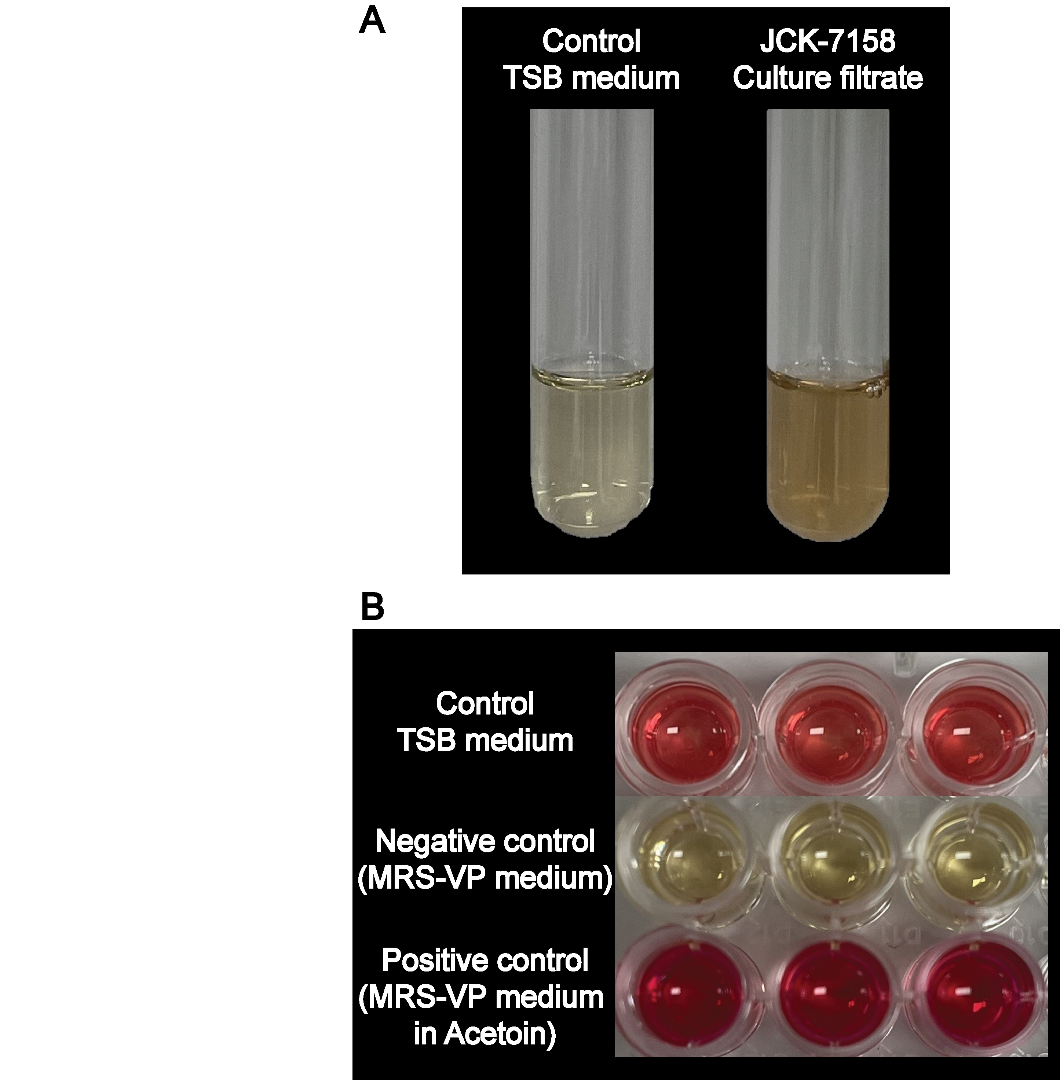
**

**Supplementary Figure 2.** Biochemical activities of JCK-7158. (A) Indole-3-acetic acid and (B) Acetoin.


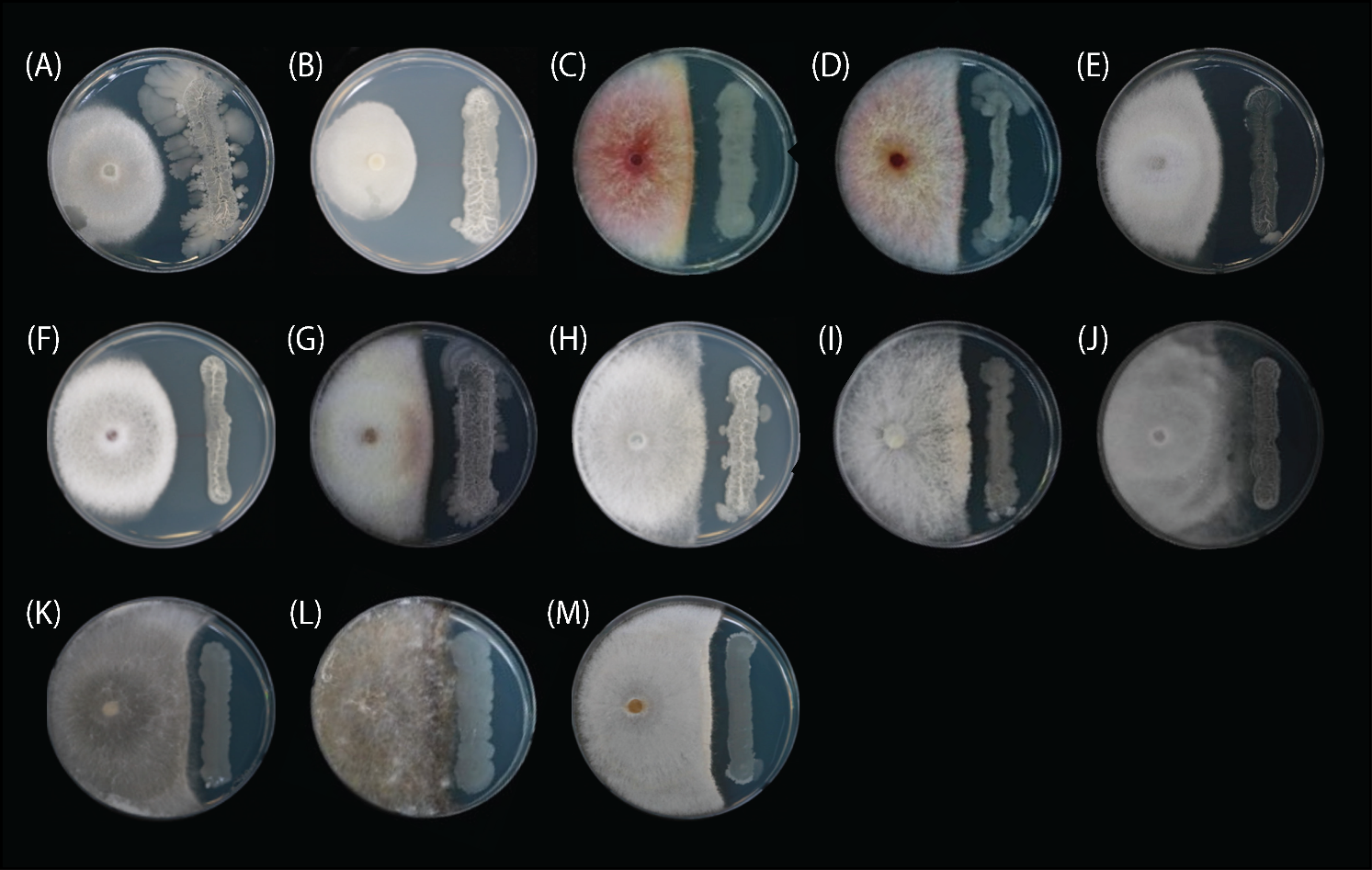


**Supplementary Figure 3.** Inhibitory activities against mycelial growth of phytopathogenic fungi by JCK-7158 in a dual culture bioassay. (A) *B. cinerea* (B) *C. coccodes* (C) *F. asiaticum* (D) *F. graminearum* (E) *F. oxysporum* f. sp. *cucumerinum* (F) *F. oxysporum* f. sp. *lycopersici* (G) *F. verticillicoides* (H) *G. graminis* (I) *P. infestans* (J) *P. ultimum* (K) *R. solani* AG 2-2 (Ⅳ) Brown patch (L) *R. solani* AG 2-2 (Ⅳ) Large patch (M) *R. solani* AG-4

**
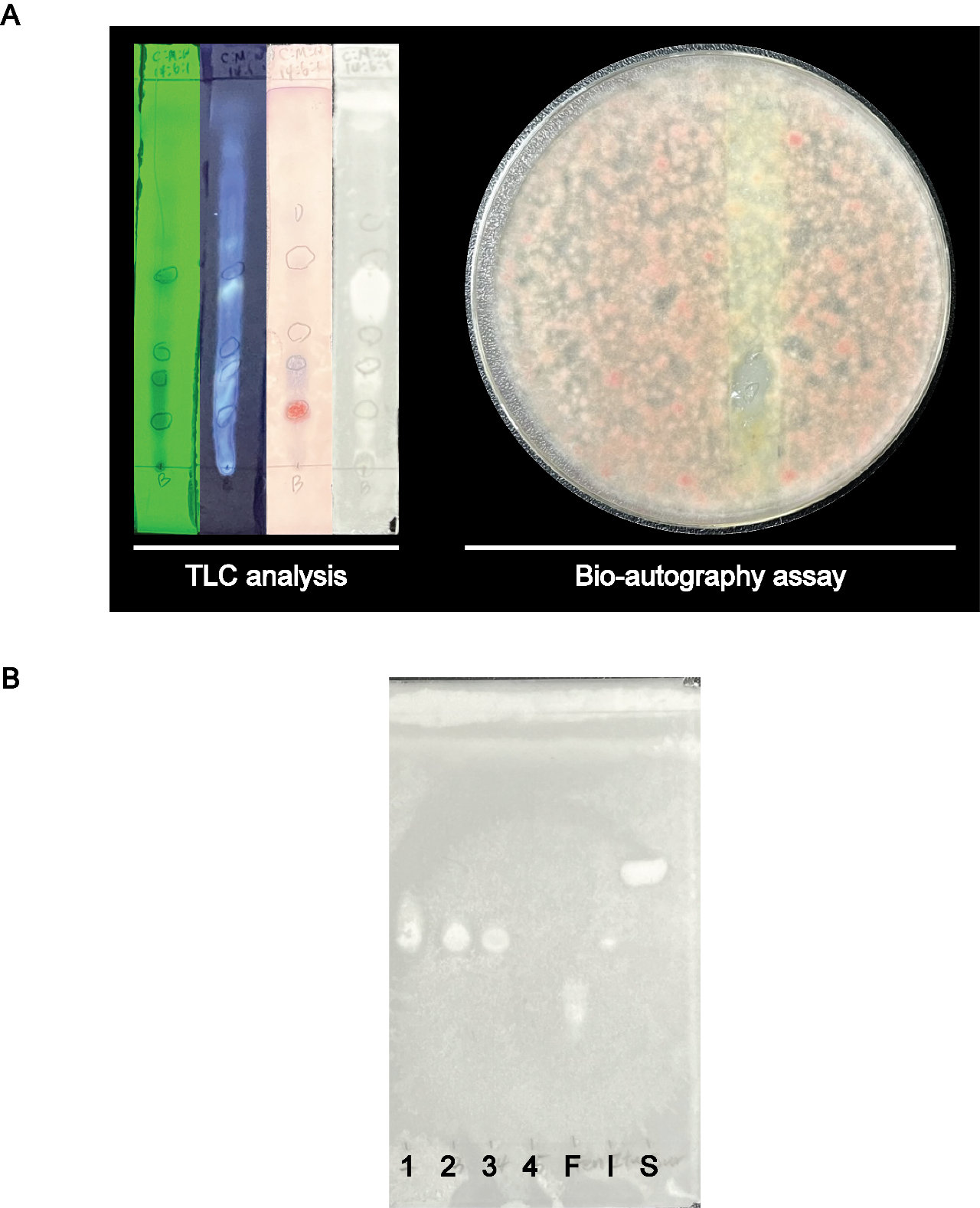
**

**Supplementary Figure 4.** Thin-layer chromatography analysis (TLC) of JCK-7158 fractions. (A) Bioautography of butanol extract of JCK-7158, and (B) TLC analysis of BF4 fractions and standards. F, fengycin; I, iturin A; S, surfactin.

**
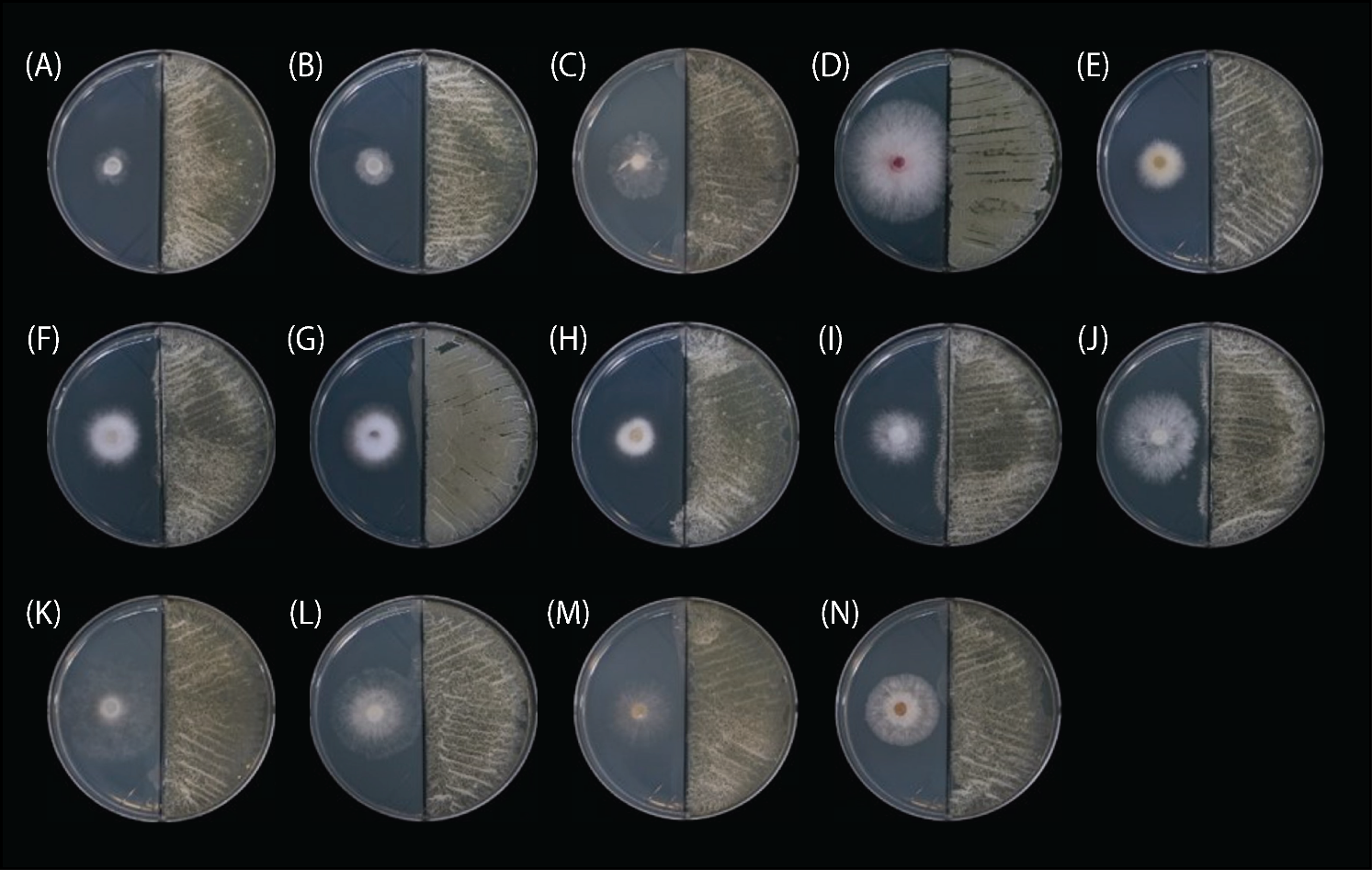
**

**Supplementary Figure 5.** Effect of the volatile compounds produced by JCK-7158 against phytopathogenic fungi in Bi-Petri Dish.

**
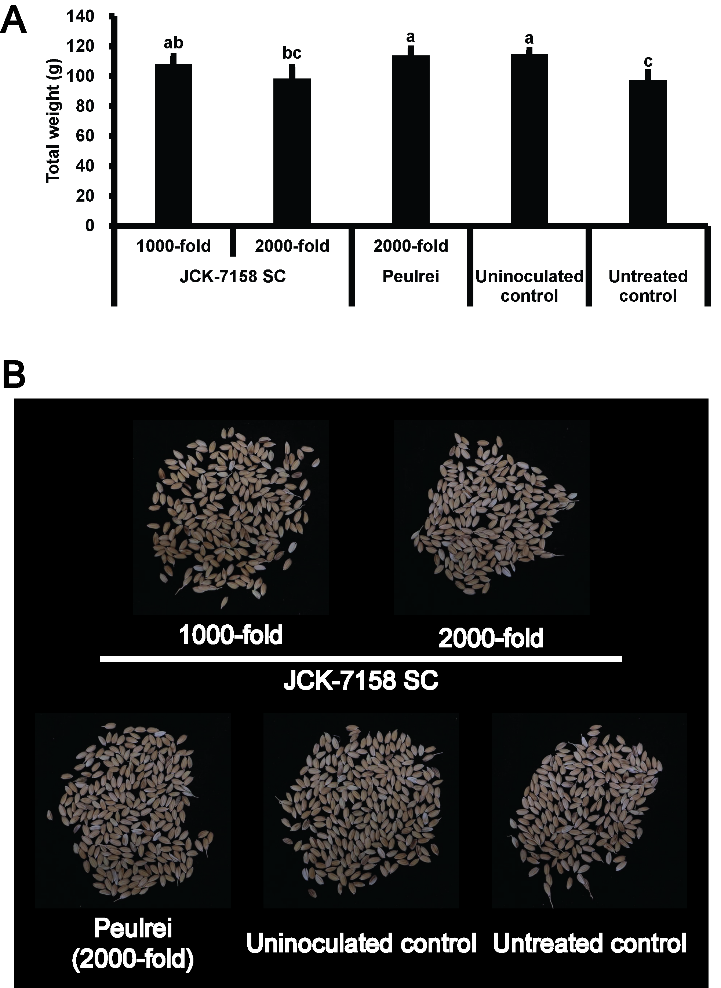
**

**Supplementary Figure 6.** Effect of JCK-7158 SC and Peulrei on total grain production and grain production of rice in the field condition. (A) Mean total grain weight of SC and Peulrei 6 weeks after inoculation, and (B) total grain production. (C) Mean grains weight removing hull of SC and Peulrei 6 weeks after inoculation and (D) production of grains with hull removed. Each value represents the means ± standard deviation of 3 plots with 30 grains per plot. Lowercase letters indicate that values are not significantly different from other values with the same letter at the P < 0.05 level, according to Duncan’s Test.

# Supplementary Tables

## Supplementary Table 1. Primer used for real-time quantitative PCR

| **Target gene** | **Forward primer (3’-5’)** | **Reverse primer (5’-3’)** |
| --- | --- | --- |
| Actin | ATCACCATCGGAGCAGAAAG | AAAAGATGGCTGGAAGAGCA |
| *OsPAL1* | TCGGCTGCGTATTCCTCA | AGTTGATGGGAAGGGGCT |
| *OsNPR1* | TGAGAGTCTACGAGGAAGGTTGC | CGTTGTCTTTCAGGAGGTGGAT |
| *PR1b* | AGAACTACGCCAGCCAGAGAAG | TTCTCGCCAAGGTTGTTCCG |
| *PR10* | CACCATCTACACCATGAAGC | AGCACATCCGACTTTAGGAC |
| *LOX* | GATGGCGGTGCTCGACGTGCT | GCACCTGTTCTTGAGCTTTCTAT |
| *OsACS1* | GAACCTGTCGTCGACGCCGA | AAAGCCGGGAGGCCGTGGT |
| *AOX1a* | CTTCGCATCGGACATCCATTA | TCCTCGGCAGTAGACAAACATC |
| *CAT* | AGGCAAGATCGTTTTCTCCA | GCGACCAGTAGGAGATCCAG |

**Supplementary Table 2.** Minimum inhibitory concentration values of BF4-1 against various phytopathogenic fungi

| **Phytopathogenic fungi** | **MIC value (ppm)** |
| --- | --- |
|  | **BF4-1** |
| *Botrytis cinerea* | 125 |
| *Botrytis cinerea* | 62.5 |
| *Clarireedia jacksonii* | 31.25 |
| *Colletotrichum coccodes* | 125 |
| *Fusarium asiaticum* | 125 |
| *Fusarium graminearum* | - |
| *Fusarium oxysporum* f. sp. *cucumerinum* | - |
| *Fusarium oxysporum* f. sp. *lycopersici* | - |
| *Fusarium verticillicoides* | 125 |
| *Gaeumannomyces graminis* | 125 |
| *Phytophthora infestans* | 62.5 |
| *Rhizoctonia solani* AG 2-2 (Ⅳ) Large patch | 62.5 |
| *Rhizoctonia solani* AG-4 | 62.5 |

**Supplementary Table 3.** List of volatile organic compounds produced by JCK-7158 through gas chromatography-mass spectrometry.

| **Retention time (min)** | **Relative peak area (%)** | **Possible compound** |
| --- | --- | --- |
| 4.158 | 4.36 | 5-Methylhexan-2-one |
| 4.7 | 24.06 | Heptan-2-one |
| 5.095 | 15.94 | 2,5-Dimethylpyrazine |
| 5.77 | 34.89 | 6-Methylheptan-2-one |
| 5.93 | 20.75 | 5-methyl-2-heptanone |
